# Targeting inflammation with chimeric antigen receptor macrophages using a signal switch

**DOI:** 10.1038/s41551-025-01387-8

**Published:** 2025-05-07

**Authors:** Qi Cao, Yiping Wang, Jianwei Chen, Ruifeng Wang, Titi Chen, Brian Gloss, Scott A. Read, Xuerong Wang, Vincent W. S. Lee, Leighton Clancy, Natasha M. Rogers, Stephen I. Alexander, Guoping Zheng, Di Yu, David C. H. Harris

**Affiliations:** 1 Centre for Transplant and Renal Research, Westmead Institute for Medical Research, The University of Sydney, Sydney, New South Wales Australia; 2 Department of Renal Medicine, Westmead Hospital, Sydney, New South Wales Australia; 3 Westmead Research Hub, Westmead Institute for Medical Research, The University of Sydney, Sydney, New South Wales Australia; 4 Storr Liver Centre, Westmead Institute for Medical Research, The University of Sydney, Sydney, New South Wales Australia; 5 Blood Transplant and Cell Therapies Laboratory, NSW Health Pathology-ICPMR Westmead, Sydney, New South Wales Australia; 6 Bone Marrow Transplant & Cell Therapies, Westmead Institute for Medical Research, The University of Sydney, Sydney, New South Wales Australia; 7 Centre for Kidney Research, Children’s Hospital at Westmead, Sydney, New South Wales Australia; 8 Faculty of Medicine, The University of Queensland Diamantina Institute, St Lucia, Queensland Australia

**Keywords:** Translational immunology, Experimental models of disease, Monocytes and macrophages

## Abstract

Chimeric antigen receptor (CAR) T-cell immunotherapy has shown great success in clinical cancer, bringing hope to apply CAR strategies to other clinical settings. Here we developed a CAR macrophage (CAR-M) that recognizes the major inflammatory molecule tumour necrosis factor (TNF) and activates an intracellular IL-4 signalling pathway, thereby programming engineered macrophages for an anti-inflammatory function. CAR-M therapy has exhibited efficacy in mouse models of both acute and chronic inflammatory diseases. In kidney ischaemia reperfusion injury (IRI), infused CAR-Ms switched to an anti-inflammatory phenotype in inflamed kidney and attenuated kidney IRI. The anti-inflammatory phenotype of infused CAR-Ms switched off during the recovery phase of kidney IRI, coinciding with the disappearance of TNF. In Adriamycin-induced nephropathy, a model of chronic inflammatory disease, infused CAR-Ms maintained an anti-inflammatory phenotype for several weeks in response to sustained high levels of TNF and improved kidney function and structure. CAR-Ms also effectively reduced tissue injury in another organ, the liver. Human anti-TNF CAR-Ms exhibit anti-inflammatory phenotype and function in response to TNF. The CAR-M design, using signal switching, holds promise for the treatment of a broad range of acute and chronic inflammatory diseases.

## Abstract (web summary)

Anti-TNF CAR macrophages show anti-inflammatory efficacy in both acute and chronic inflammatory disease mouse models.

## Main

Inflammation plays a pivotal role in many diseases, and failure to resolve inflammation is a pivotal pathological mechanism that drives disease progression, ultimately leading to severe consequences such as end organ failure^1^. Currently, available drugs targeting inflammation, especially in chronic diseases, are toxic and have limited efficacy.

Macrophages, strategically located throughout the body, play a crucial role in maintaining internal balance by clearing invading pathogens and harmful substances^2,3^. Their notable phagocytic ability, regenerative capacity and role as inflammatory regulators make them promising sources for cellular therapy. We and others have shown that administered anti-inflammatory macrophages (M2) can reduce injury in various disease models^4–9^. However, the efficacy of M2 is limited by their phenotypic plasticity that can result in pathogenic transformation upon exposure to inflammatory microenvironments in diseased organs^10–12^. Ongoing clinical trials are investigating the potential of macrophage-based cell therapy in conditions such as ischaemic stroke, cardiomyopathy, liver cirrhosis, kidney failure and anal fissure^13,14^. Enhancing the phenotypic stability of M2 is crucial for advancing macrophage therapy.

Chimeric antigen receptor (CAR) T-cell therapy is a very promising immunotherapeutic breakthrough which has achieved great success in the treatment of B-cell lymphomas and other tumours^15,16^. The CAR T-cell approach has been extended to natural killer cells and macrophages in recent years^17,18^. The remarkable advances achieved by CAR T-cell therapy in the field of oncology have sparked a surge of interest that extends far beyond its original scope into the fields of autoimmunity and fibrotic diseases^19,20^. In this Article, we developed CAR macrophages (CAR-Ms) designed to respond to inflammatory cytokines for the treatment of inflammatory diseases in various organs. Unlike traditional CARs that augment immune cell functions to enhance immunity, this type of CAR is unique in converting immune effector responses to an immunosuppressive response. When triggered by inflammatory cytokines within injured tissue, CAR-Ms induce anti-inflammatory and wound-healing responses, effectively resolving inflammation and reducing tissue injury.

The central aim of this study was to examine the efficacy of CAR-Ms against inflammation in relevant models of kidney and liver diseases. We have demonstrated that human and mouse CAR-Ms can dynamically switch phenotype and function to become anti-inflammatory in response to the inflammatory molecule tumour necrosis factor (TNF). Infused CAR-Ms attenuated inflammation and injury in both acute and chronic inflammatory kidney diseases, as well as inflammatory liver disease.

## Results

### Generation and characterization of CAR-Ms

**Fig. 1:**
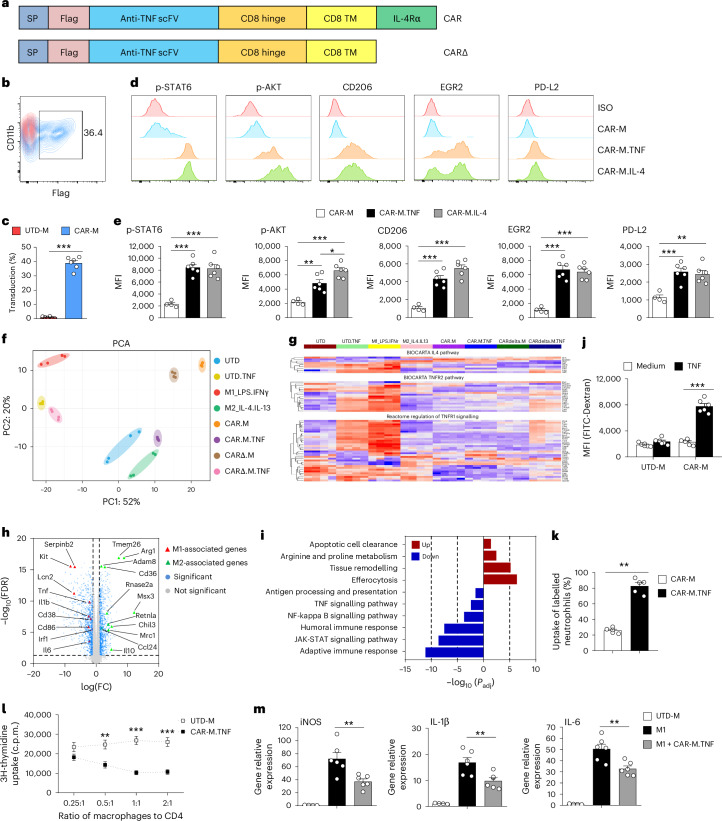
Generation and characterization of CAR-Ms. **a**, Schematic representation of the anti-TNF CAR construct design for macrophages. The CAR contains a Flag tag epitope, a scFv antibody specific for TNF, the hinge and transmembrane domains of mouse CD8α and a mouse IL-4 receptor intracellular domain. **b**, Representative flow cytometric analysis of transduction efficiency (Flag-tag expression) on macrophages 3 days after transduction with CAR (blue, CAR-M; red, UTD-M). **c**, Quantitative analysis of the percentage of Flag+ cells on CAR-M and UTD-M. Data shown are the mean ± s.e.m. (*n* = 6 per group). ***P < 0.001. **d**, Representative flow cytometric analysis of mouse M2 markers p-STAT6, p-AKT, CD206, EGR2 and PD-L2 on CAR-M in response to TNF or IL-4. ISO, isotype. **e**, Flow cytometric analysis of mean fluorescence intensity (MFI) of M2 markers. Data shown are the mean ± s.e.m. (*n* = 4–6 per group) and are representative of 3 independent experiments. *P < 0.05, **P < 0.01, ***P < 0.001. **f**, Gene expression principal component analysis clustering from UTD, UTD.TNF, M1, M2, CAR-M, CAR-M.TNF, CARΔ-M and CARΔ-M.TNF. *n* = 4 per group. **g**, Scaled expression heat map of genes families in important pathways (accessed using molecular signatures database (MSIGDB)). TNFR, tumor necrosis factor receptor. **h**, Volcano plot of differentially expressed genes in CAR-M.TNF versus CAR-M. Blue indicates *P*_adj_ < 0.05 and log_2_(fold change) >1 or <−1. Red triangles indicate M1-associated genes, and green triangles indicate M2-associated genes (*n* = 4 per group). Statistical significance was calculated using the QLFtest for DGE data. **i**, Gene Ontology and KEGG pathway analysis results of upregulated and downregulated pathways in CAR-M.TNF versus CAR-M. **j**, UTD-M and CAR-M were cocultured with FITC-labelled dextran in the presence or absence of TNF for 45 min. The uptake of fluorescent beads (MFI) was determined by flow cytometry. Data shown are the mean ± s.e.m. (*n* = 6 per group) and are representative of 2 independent experiments. ***P < 0.001. **k**, The UTD-Ms and CAR-M.TNF were incubated with PKH26 Red-labelled apoptotic cells for 3 h. The percentage of F4/80+ macrophages that had incorporated the apoptotic neutrophils was determined by flow cytometry. Data shown are the mean ± s.e.m. (*n* = 5 per group) and are representative of 3 independent experiments. **P < 0.01. **l**, Inhibition of CD4 T-cell proliferation was examined with various dosages of UTD-Ms and CAR-M.TNF. c.p.m, counts per minute. Data shown are the mean ± s.e.m. (*n* = 5 per group) and are representative of 2 independent experiments. **P < 0.01, ***P < 0.001 versus UTD-M. **m**, M1 macrophages were cocultured with TNF-activated CAR-Ms for 24 h. The mRNA expression of iNOS, IL-1β and IL-6 of M1 was examined by qPCR. Data shown are the mean ± s.e.m. (*n* = 4–6 per group) and are representative of 3 independent experiments. **P < 0.01. Source data

**Extended Data Fig. 1:**
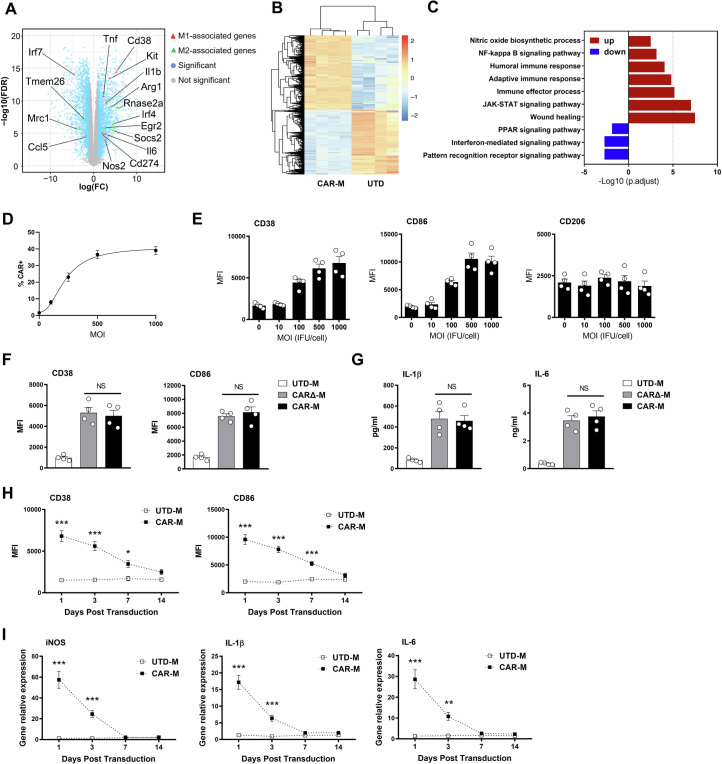
Lentivirus transduction led to a transient pro-inflammatory macrophage phenotype. (**A**) Volcano plot of differentially expressed genes in CAR-M versus UTD. Blue indicates Padj < 0.05 and log2 fold change >1 or <−1. Red triangles indicate significant M1-associated genes and green triangles indicate significant M2-associated genes (n = 4 per group). Statistical significance was calculated using the QLFtest for DGE data. (**B**) Hierarchical clustering of differentially expressed genes from UTD or CAR-M (n = 4 per group), 48 hours post transduction. The heatmap shows log2 fold-change in gene expression relative to UTD. (**C**) GO and KEGG pathway analysis results of up- and down-regulated pathways in CAR-M, compared to UTD. (**D**) Mouse bone marrow derived macrophages were transduced with CAR at MOIs of 0, 100, 250, 500, or 1000 IFU. CAR expression on macrophages correlated with MOI. Data shown are the mean ± SEM (n = 4 per group). Correlation was determined via nonlinear regression and Pearson. (**E**) Flow cytometry analysis of mouse M1 markers CD38 and CD86 and M2 marker CD206 in response to transduction with increasing MOIs of CAR by FACS. Data shown are the mean ± SEM (n = 4 per group) and are representative of 2 independent experiments. (**F**) Surface expression of M1 markers CD38 and CD86 after transduction with equivalent MOIs of CARΔ and CAR. (**G**) Production of IL-1β and IL-6 after transduction with equivalent MOIs of CARΔ and CAR. Data shown are the mean ± SEM (n = 4 per group) and are representative of 2 independent experiments. (**H** and **I**) Transient expression of M1 markers (CD38, CD86, iNOS, IL-1β and IL-6) on CAR-Ms during 14 days of in vitro culture. Data shown are the mean ± SEM (n = 4 per group). NS: not significant, *P < 0.05, **P < 0.01, ***P < 0.001 vs UTD. MOI: multiplicity of infection. Source data

To achieve CAR-mediated redirection of macrophage phenotype, we created a CAR construct with an anti-TNF single-chain fragment variable (scFv) as the extracellular domain linked to the intracellular domain of interleukin-4 (IL-4) receptor alpha (IL-4Rα) (Fig. 1a). This unique CAR design allowed the anti-TNF scFv to specifically bind TNF, triggering a signal switch towards an anti-inflammatory response by connecting to the IL-4Rα domain. The adenovirus Ad5f35 has been shown to efficiently transduce primary human macrophages. However, the prolonged pro-inflammatory (M1) macrophage phenotype induced by adenoviral infection raises substantial doubts about its application in inflammatory diseases^18^. Here we engineered an anti-TNF CAR into a lentivirus vector and demonstrated that lentivirus-transduced mouse bone-marrow-derived macrophages (BMDMs) expressed CAR efficiently (Fig. 1b,c). Lentivirus transduction led to the induction of M1-associated genes including inducible nitric oxide synthase (iNOS), TNF, interleukin-1 beta (IL-1β) and CD38, along with activation of inflammatory pathways, including those of nuclear factor kappa B (NF-κB) signalling, Janus kinase-signal transducers and activators of transcription (JAK-STAT) signalling and immune effector processes. It is worth noting that after lentivirus infection, some M2-associated genes and pathways were also induced (Extended Data Fig. 1a–c), which was not seen with adenovirus transduction. There was a dose-dependent expression of co-stimulatory ligands in response to lentivirus transduction (Extended Data Fig. 1d,e). Induction of co-stimulatory ligands and inflammatory cytokines was equivalent between CAR-M and CARΔ (a truncated CAR lacking the IL-4Rα intracellular domain)-M, demonstrating that the pro-inflammatory phenotype is induced by the viral transfection process rather than CAR expression in macrophages (Extended Data Fig. 1f,g). The expression of co-stimulatory ligands and inflammatory cytokines was maintained for only 3 days (Extended Data Fig. 1h,i), suggesting that lentivirus transduction induces a transient pro-inflammatory macrophage phenotype, suitable for inflammatory disease applications.

**Extended Data Fig. 2:**
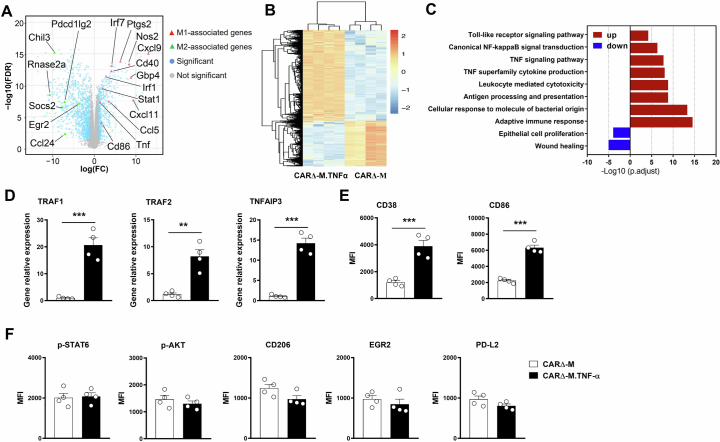
TNF-α did not induce M2 phenotype in macrophages transduced with a truncated CAR (CARΔ). (**A**) Volcano plot of differentially expressed genes in CARΔ-M + TNF-α versus CARΔ-M. Blue indicates Padj < 0.05 and log2 fold change >1 or <−1. Red triangles indicate significant M1-associated genes and green triangles indicate significant M2-associated genes (n = 4 per group). Statistical significance was calculated using the QLFtest for DGE data. (**B**) Hierarchical clustering of differentially expressed genes from CARΔ-M + TNF-α versus CARΔ-M (n = 4 per group). The heatmap shows log2 fold-change in gene expression relative to CARΔ-M. (**C**) GO and KEGG pathway analysis results of up- and down-regulated pathways in CARΔ-M.TNF-α, compared to CARΔ-M. (**D**) Quantitative PCR analysis of TNF-α signalling pathway in CARΔ-M in response to TNF-α. (**E** and **F**) Flow cytometric analysis of mouse M1 markers (CD38 and CD86) and M2 markers (p-STAT6, p-AKT, CD206, EGR2 and PD-L2) on CARΔ-M in response to TNF-α. Data shown are the mean ± SEM (n = 4 per group) and are representative of 3 independent experiments. **P < 0.01, ***P < 0.001. Source data

**Extended Data Fig. 3:**
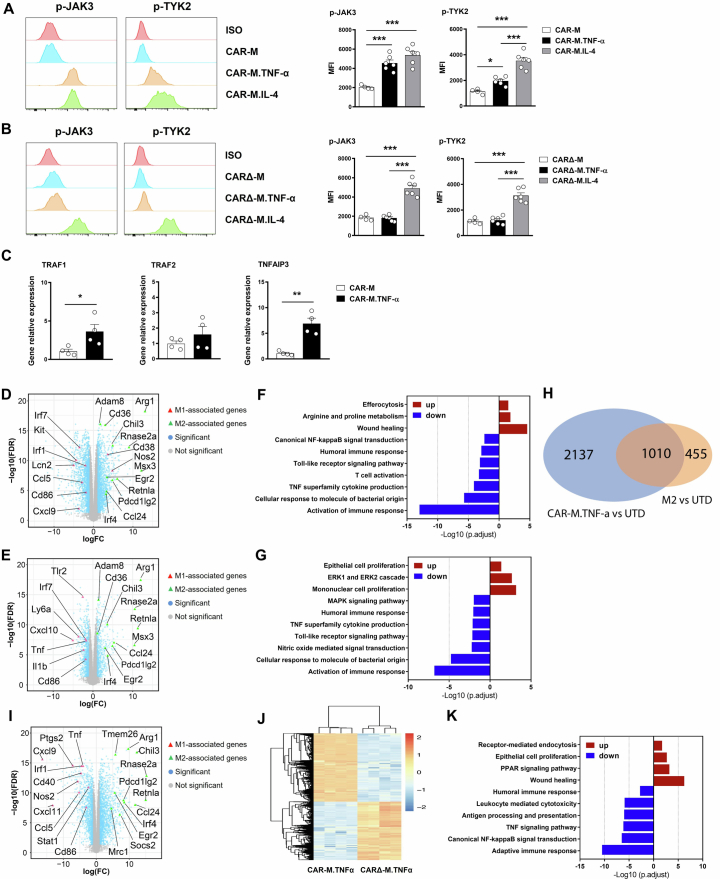
Characterization of CAR-M. (**A** and **B**) Representative flow cytometric analysis of IL-4 signalling pathway p-JAK3 and p-TYK2 on CAR-M or CARΔ-M in response to TNF-α or IL-4. Data shown are the mean ± SEM (n = 4-6 per group). *P < 0.05, ***P < 0.001. (**C**) Quantitative PCR analysis of TNF-α signalling pathway on CAR-M in response to TNF-α. Data shown are the mean ± SEM (n = 4 per group) and are representative of 2 independent experiments. **P < 0.01. (**D** and **E**) Volcano plot of differentially expressed genes in CAR-M.TNF-α versus UTD and M2 versus UTD. Blue indicates Padj < 0.05 and log2 fold change >1 or <−1. Red triangles indicate significant M1-associated genes and green triangles indicate significant M2-associated genes (n = 4 per group). Statistical significance was calculated using the QLFtest for DGE data. (**F** and **G**) GO and KEGG pathway analysis results of up- and down-regulated pathways in CAR-M.TNF-α versus UTD and M2 versus UTD. (**H**) Venn diagram show the overlapping differently expressed genes from CAR-M.TNF-α versus UTD and M2 versus UTD. (**I**) Volcano plot of differentially expressed genes in CAR-M.TNF-α versus CARΔ-M.TNF-α. (**J**) Hierarchical clustering of differentially expressed genes from CAR-M.TNF-α versus CARΔ-M.TNF-α (n = 4 per group). The heatmap shows log2 fold-change in gene expression relative to CARΔ-M.TNF-α. (**K**) GO and KEGG pathway analysis results of up- and down-regulated pathways in CAR-M.TNF-α, compared to CARΔ-M.TNF-α. Source data

We next examined CAR-mediated redirection of macrophage phenotype in macrophages expressing intact CAR or CARΔ. CAR activity on macrophages was evaluated by the degree of phosphorylation of signal transducer and activator of transcription factor 6 (p-STAT6) and phosphorylation of serine/threonine protein kinase (p-AKT), the downstream transcription factors activated by the intracellular domain of IL-4Rα. Treatment with TNF resulted in a rapid increase in p-STAT6 and p-AKT expression specifically in CAR-Ms, but not in CARΔ-Ms. Consistently, TNF-treated CAR-Ms (CAR-M.TNF), but not TNF-treated CARΔ-Ms (CARΔ-M.TNF), exhibited a significant upregulation of M2 markers, including CD206, early growth response 2 (EGR2) and programmed cell death ligand 2 (PD-L2) (Fig. 1d,e and Extended Data Fig. 2). Induction of IL-4 downstream signalling pathways and M2 surface markers was equivalent between CAR-M.TNF and IL-4-treated CAR-M. Furthermore, when untransduced macrophages (UTD), UTD.TNF, M1, M2, CAR-M, CAR-M.TNF, CARΔ-M and CARΔ-M.TNF were subjected to non-biased principal component analysis, CAR-M.TNF clustered towards the M2 phenotype and away from M1 phenotype, whereas TNF-treated UTD and CARΔ-Ms clustered towards the M1 phenotype and away from M2 phenotype (Fig. 1f). TNF stimulation selectively induced the IL-4 downstream signalling pathway in CAR-Ms, whereas it exclusively triggered the TNF downstream signalling pathway in CARΔ-Ms and UTD-Ms (Fig. 1g, Extended Data Figs. 2 and 3 and Supplementary Fig. 1). Specifically, TNF stimulation in CAR-Ms led to the upregulation of M2-associated genes and pathways related to tissue remodelling and efferocytosis. Concurrently, there was a downregulation of M1-associated genes and pathways, including NF-kappa B signalling pathway and JAK-STAT signalling pathway (Fig. 1h,i). However, TNF stimulation of CARΔ-Ms and UTD-Ms resulted in upregulation of M1-associated genes and pathways and downregulation of M2-associated genes and pathways (Extended Data Fig. 2 and Supplementary Fig. 1). The IL-4 receptor complex comprises two proven types^21^: type 1 IL-4R constituted by IL-4Rα and IL-2Rγc chains and type 2 IL-4R constituted by IL-4Rα and IL-13Rα1 chains. Compared with IL-4 stimulation, TNF fully activated p-JAK3 (IL-2Rγc chain) but only partially activated p-TYK2 (IL-13Rα1 chain) on CAR-Ms, indicating TNF mainly activates type 1 IL-4 receptor complex through CAR on macrophages (Supplementary Fig. 1a,b). CAR-M.TNF and M2 macrophages showed similar M2 phenotypes, as evidenced by shared signature genes and pathways (Extended Data Fig. 3d–h). Compared to CARΔ-M.TNF, CAR-M.TNF showed upregulation of M2-associated genes and pathways, along with downregulation of M1-associated genes and pathways (Extended Data Fig. 3i–k). CAR-M.TNF exhibited heightened phagocytic activity and enhanced clearance of dead cells compared to UTD-Ms or CAR-Ms, suggesting an augmented wound-healing capacity (Fig. 1j,k). The immunosuppressive function of CAR-M was assessed through coculture experiments with CD4 T cells and M1 macrophages. CAR-M.TNF significantly suppressed T-cell proliferation and deactivated M1 macrophages (Fig. 1l,m). These findings collectively demonstrate that anti-TNF CAR redirects macrophage responses away from endogenous TNF signalling towards immunosuppressive and tissue-repair pathways. The relative responsiveness of CAR T cells to soluble antigens has posed a substantial challenge. A recent study indicates that the dimeric form of soluble antigens, such as dimeric cytokine transforming growth factor-beta (TGF-β), is necessary to trigger CAR signalling^22^. In this study, we present evidence demonstrating that soluble trimeric cytokine TNF can activate our CAR. It is worth noting that immobilized TNF induced stronger CAR activation compared to soluble TNF, as evidenced by a significant increase in p-STAT6 and other M2 markers (Supplementary Fig. 2).

### CAR-Ms switched to an anti-inflammatory phenotype in inflamed kidney and attenuated kidney ischaemia reperfusion injury

**Fig. 2:**
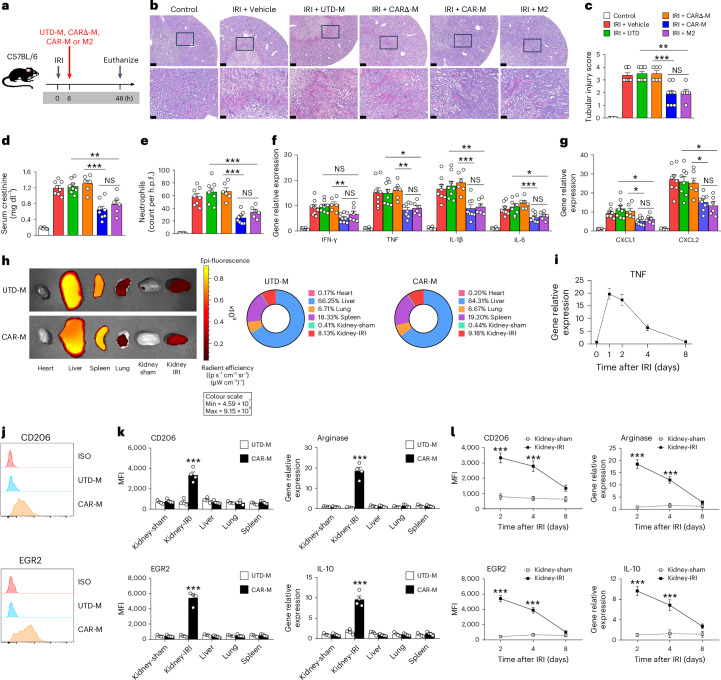
Evaluation of protective effect of CAR-M in acute kidney injury model. **a**, C57BL/6 mice were treated with CD45.1^+^ UTD-M, CARΔ-M, CAR-M or M2 at 6 h after bilateral IRI. Mice were euthanized at 48 h after IRI. **b**, Representative PAS-stained sections of kidney outer medulla from IRI mice treated with UTD-M, CARΔ-M, CAR-M or M2. Each panel shows low (top, bar = 250 µm) and high (bottom; corresponding to the boxed area, bar = 50 µm) power view. **c**, Semiquantitative assessment of tubular injury. **P < 0.01, ***P < 0.001. **d**, Serum creatinine levels were assessed in these mice. **P < 0.01, ***P < 0.001. **e**, Number of Gr-1^+^ neutrophils was assessed by immunofluorescence staining in the outer medulla of the kidney. h.p.f., high-power field. ***P < 0.001. **f**,**g**, The mRNA levels of pro-inflammatory cytokines (**f**) and chemokines (**g**) in the kidneys. Data shown are the mean ± s.e.m. (*n* = 6–8 per group). NS, not significant; *P < 0.05; **P < 0.01; ***P < 0.001. **h**, The biodistribution of XenoLight DiR-labelled UTD-M or CAR-M in unilateral IRI model. The IRI mice were euthanized, and organs were explanted for ex vivo imaging 48 h after injection. *n* = 4–5 mice per treatment group. **i**, The mRNA level of TNF in the kidneys over the course of kidney IRI. Data shown are the mean ± s.e.m. (*n* = 4 per group). **j**,**k**, CD45.1^+^ UTD-Ms and CD45.1^+^ CAR-Ms were sorted from various organs at 48 h after kidney IRI. Representative flow cytometric analysis of M2 markers CD206 and EGR2 on transfused UTD-M and CAR-M in kidney with IRI (**j**). The M2-like phenotype of transfused macrophages from various organs (**k**) was assessed by flow cytometry (CD206 and EGR2) and qPCR (Arginase and IL-10). Data shown are the mean ± s.e.m. (*n* = 4 per group). ***P < 0.001. **l**, The M2-like phenotype of transfused macrophages over the course of IRI was assessed by flow cytometry and qPCR. Data shown are the mean ± s.e.m. (*n* = 4 per group). ***P < 0.001 versus Kidney-sham. Source data

**Extended Data Fig. 4:**
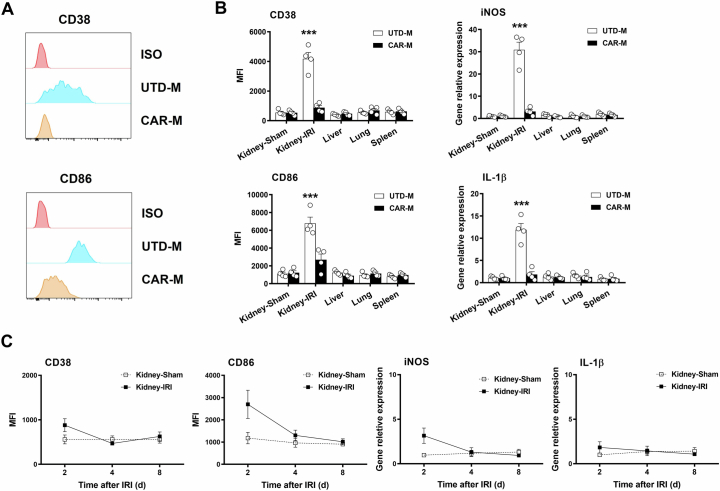
CAR-Ms did not switch to a pro-inflammatory phenotype in inflamed kidney of IRI mice. (**A** and **B**) CD45.1 + UTD-Ms and CD45.1 + CAR-Ms were sorted from various organs at 48 hours after kidney IRI. Representative flow cytometric analysis of M1 markers CD38 and CD86 on transfused UTD-M and CAR-M in kidney with IRI (**A**). The M1-like phenotype of transfused macrophages from various organs (**B**) was assessed by flow cytometry (CD38 and CD86) and quantitative PCR (iNOS and IL-1β). Data shown are the mean ± SEM (n = 4 per group). ***P < 0.001. (**C**) The M1-like phenotype of transfused macrophages over the course of IRI was assessed by flow cytometry and quantitative PCR. Data shown are the mean ± SEM (n = 4 per group). Source data

We next tested the protective effect of CAR-M in vivo using an acute kidney injury model, kidney ischaemia reperfusion injury (IRI). Mice were treated with CD45.1^+^ UTD-Ms, CARΔ-Ms, CAR-Ms or M2 at 6 h after kidney IRI (Fig. 2a). CAR-M-treated mice killed 48 h after IRI showed less tubular injury, lower serum creatinine level, less neutrophil infiltration and reduction of inflammatory cytokine/chemokine expression in kidney, indicating that the CAR-M triggered by inflammatory cytokine TNF can effectively treat kidney IRI (Fig. 2b–g). CAR-M and M2 exhibit equivalent protective effects in the kidney IRI model. To evaluate the ability of UTD-M or CAR-M to traffic to inflamed kidney, fluorescently labelled macrophages were administered in a unilateral IRI model, and the relative signal in the heart, liver, lung, spleen and both kidneys was evaluated in explanted whole-organ tissue on day 2 (Fig. 2h). Both UTD-Ms and CAR-Ms tended to distribute to inflamed kidney, but not healthy kidney. In all mice, infused macrophages accumulated more in normal liver than in other organs. Lentiviral transduction did not affect the trafficking capability of macrophages. In addition, CAR-Ms did not cause damage to other normal tissues such as the liver, lung, heart and spleen in vivo (Supplementary Fig. 3). The TNF level in inflamed kidney was significantly increased at day 1 and gradually decreased from day 2 to day 8 in IRI mice (Fig. 2h,i). CAR-Ms, but not UTD-Ms, transitioned to an anti-inflammatory phenotype in response to elevated TNF within the inflamed kidney at day 2 after kidney IRI (Fig. 2j,k). It is worth noting that this transition did not occur in healthy kidneys or other organs. In contrast to UTD-Ms, infused CAR-Ms did not convert to a pro-inflammatory phenotype within the inflamed kidney (Extended Data Fig. 4). A pivotal question is the duration of the M2-like phenotype of CAR-M in situ. The anti-inflammatory phenotype observed in infused CAR-Ms subsided during the recovery phase of kidney IRI, paralleling the disappearance of the inflammatory cytokine TNF (Fig. 2l).

**Extended Data Fig. 5:**
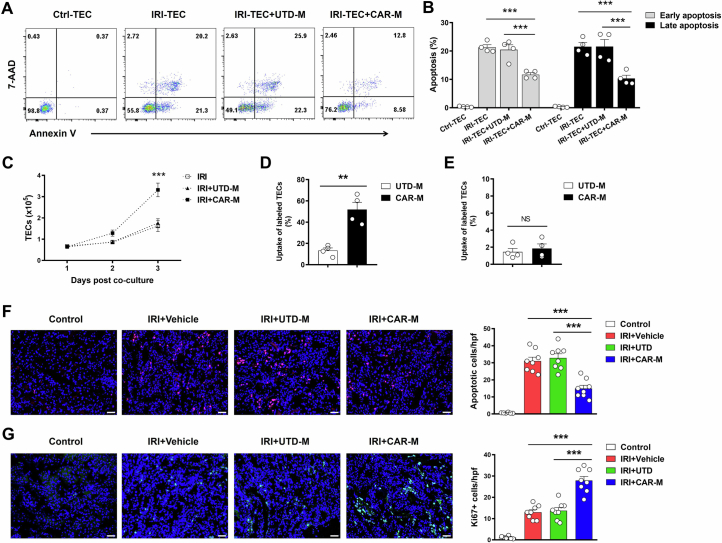
CAR-Ms reduced TEC apoptosis and promoted TEC proliferation. UTD-Ms or TNF-α-treated CAR-Ms were cocultured with ischemic kidney TECs (IRI-TECs) for 1–3 days. TECs were exposed to serum-free K1 medium alone as the nonischemic control (Ctrl-TEC). (**A**) Representative FACS analysis of apoptosis in TECs after 1-day coculture. (**B**) Frequency of early apoptosis (Annexin V + 7AAD- cells) and late apoptosis (Annexin V + 7AAD+ cells) in TECs after 1-day coculture. Data shown are the mean ± SEM (n = 4 per group) and are representative of 2 independent experiments. ***P < 0.001. (**C**) Numbers of kidney TECs were counted at each time point after coculture. Data shown are the mean ± SEM (n = 6 per group) and are representative of 3 independent experiments. ***P < 0.001. (**D** and **E**) The UTD-Ms and TNF-α-treated CAR-Ms were incubated with PKH26 Red-labeled ischemic kidney TECs (**D**) or nonischemic normal TECs (**E**) for 4 h. The percentage of macrophages that had incorporated the TECs was determined by flow cytometry. Data shown are the mean ± SEM (n = 4 per group) and are representative of 2 independent experiments. NS: not significant, **P < 0.01. (**F**) Kidney tubular epithelial cell apoptosis was measured by terminal deoxynucleotidyl transferase–mediated dUTP nick end-labeling assay on the representative sections of the kidney. (**G**) Kidney tubular epithelial cell proliferation was assessed by immunofluorescence staining for Ki67 on representative sections of kidney. Bar=50 µm. Data shown are the mean ± SEM (n = 6-8 per group). ***P < 0.001. Source data

To understand the mechanism behind the preferential conversion of CAR-M to an anti-inflammatory phenotype within the inflamed kidney, where numerous inflammatory cytokines are in high concentration, we examined phenotypic plasticity by subjecting UTD-M or CAR-M to M1-inducing inflammatory cytokines in vitro. Stimulation with IFNγ or a mixture of inflammatory cytokines (mimicking the in vivo inflammatory environment) led to the upregulation of typical M1 markers in both UTD-Ms and CAR-Ms. It is worth noting that when TNF was introduced into the coculture system, it attenuated the effects of M1-inducing cytokines on CAR-Ms (Supplementary Fig. 4). Tubular epithelial cell (TEC) injury is a prominent feature of kidney IRI. In contrast to UTD-Ms, CAR-M.TNF not only markedly reduced apoptosis in ischaemic TECs but also enhanced TEC proliferation in vitro (Extended Data Fig. 5a–c). CAR-M.TNF enhanced clearance of apoptotic TECs compared to UTD-Ms, but not that of healthy TECs (Extended Data Fig. 5d,e). Furthermore, IRI mice treated with CAR-Ms exhibited a decrease in apoptotic TECs and an increase in TEC proliferation in vivo, suggesting that CAR-M actively promoted survival and regeneration of ischaemic tubular cells in IRI mice (Extended Data Fig. 5f,g). Endogenous macrophages and regulatory T cells (T_reg_ cells) have been shown to modify the disease progression of IRI. CAR-Ms treatment reduced pro-inflammatory M1 endogenous macrophages but did not induce T_reg_ cells in the kidney of IRI mice (Supplementary Fig. 5).

### CAR-Ms did not switch to an anti-inflammatory phenotype and failed to protect against kidney injury in TNF-deficient mice with IRI

**Fig. 3:**
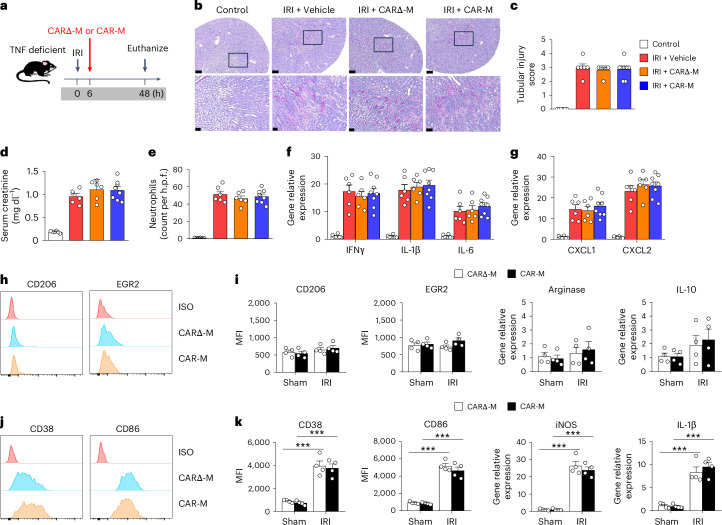
Failed renoprotection of CAR-Ms in TNF-deficient mice with IRI. **a**, TNF-deficient mice were treated with CD45.1^+^ CARΔ-M or CAR-M at 6 h after bilateral IRI. Mice were euthanized at 48 h after IRI. **b**, Representative PAS-stained sections of kidney outer medulla from IRI mice treated with CARΔ-M or CAR-M. Each panel shows low (top, bar = 250 µm) and high (bottom; corresponding to the boxed area, bar = 50 µm) power view. **c**, Semiquantitative assessment of tubular injury. **d**, Serum creatinine levels were assessed in these mice. **e**, Number of Gr-1^+^ neutrophils was assessed by immunofluorescence staining in the outer medulla of the kidney. **f**,**g**, The mRNA levels of pro-inflammatory cytokines (**f**) and chemokines (**g**) in the kidneys. Data shown are the mean ± s.e.m. (*n* = 6–8 per group). **h**,**i**, CARΔ-Ms and CAR-Ms were sorted from kidneys at 48 h after kidney IRI. Representative flow cytometric analysis of M2 markers (CD206 and EGR2 (**h**)) and M1 markers (CD38 and CD86 (**i**)) on transfused CARΔ-Ms and CAR-Ms in kidney with IRI. **j**,**k**, The M2 (**j**) and M1-like (**k**) phenotype of transfused macrophages from Sham or IRI kidney was assessed by flow cytometry and qPCR. Data shown are the mean ± s.e.m. (*n* = 4 per group). ***P < 0.001. Source data

To validate that TNF is the decisive factor inducing the therapeutic effect of the CAR-M, we used TNF-deficient mice (Supplementary Fig. 6). TNF-deficient mice were treated with CD45.1^+^ CARΔ-Ms and CAR-Ms 6 h after kidney IRI (Fig. 3a). CAR-M treatment did not show any protective effect on structural and functional kidney injury and did not reduce neutrophil infiltration or inflammatory cytokine/chemokine expression in kidney in TNF-deficient mice with IRI, indicating the TNF-dependent protective effect of CAR-Ms (Fig. 3b–g). Moreover, infused CAR-Ms did not convert to an anti-inflammatory phenotype but rather to a pro-inflammatory phenotype within the inflamed kidney of TNF-deficient mice (Fig. 3h–k). Collectively, these data further confirmed that CAR-Ms can harness the inflammatory cytokine TNF to exert their therapeutic effect.

### CAR-Ms sustained an anti-inflammatory phenotype in inflamed kidney and reduced functional and structural injury in chronic kidney disease

**Fig. 4:**
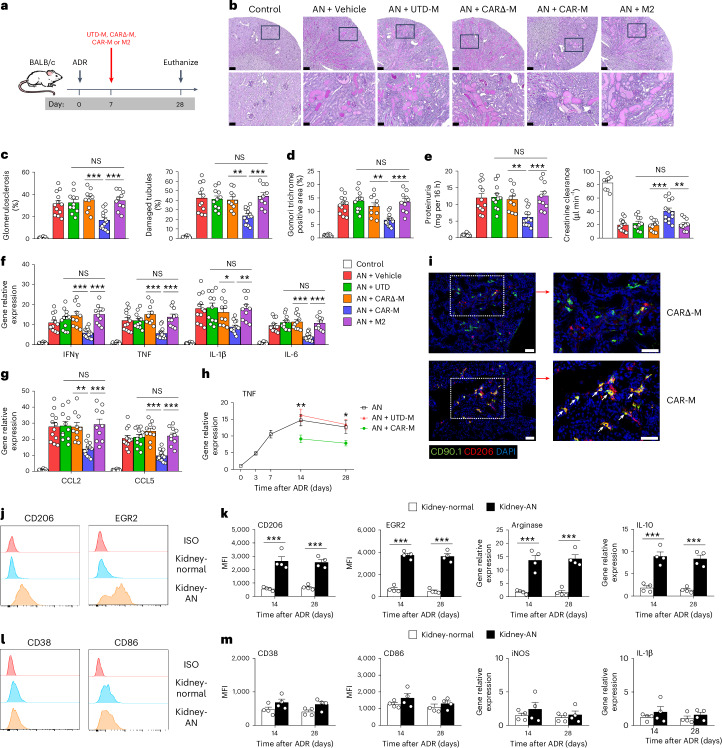
Evaluation of protective effect of CAR-M in chronic kidney disease model. **a**, BALB/c mice were treated with UTD-M, CARΔ-M, CAR-M or M2 at day 7 after ADR injection. Mice were euthanized at 28 days. **b**, Representative PAS-stained sections of kidney from AN mice treated with UTD-M, CARΔ-M, CAR-M or M2. Each panel shows low (top, bar = 250 µm) and high (bottom; corresponding to the boxed area, bar = 50 µm) power view. **c**, Quantitative assessment of glomerular sclerosis and tubular damage. **P < 0.01, ***P < 0.001. **d**, Quantitative analysis of the positive area of Gomori Trichrome staining (kidney fibrosis). **P < 0.01, ***P < 0.001. **e**, Proteinuria and creatinine clearance were assessed at day 28 after ADR injection. **P < 0.01, ***P < 0.001. **f**,**g**, mRNA levels of pro-inflammatory cytokines (**f**) and chemokines (**g**) in kidneys. Data shown are the mean ± s.e.m. (*n* = 8–13 per group). *P < 0.05, **P < 0.01, ***P < 0.001. **h**, mRNA level of TNF in the kidneys over the course of AN. Data shown are the mean ± s.e.m. (*n* = 4 per group). *P < 0.05, **P < 0.01. **i**, Immunofluorescence double staining of CD90.1 and CD206 in kidney sections of AN mice at day 28. The white arrows indicate CD90.1^+^CD206^+^ CAR-Ms. Scale bars, 50 µm. **j**,**l**, CD90.1^+^ CAR-Ms were sorted from normal kidney and AN kidney at days 14 and 28 after ADR injection. Representative flow cytometric analysis of M2 markers (CD206 and EGR2, **j**) and M1 markers (CD38 and CD86, **l**) on transfused CAR-Ms from normal kidney and AN kidney at day 14. **k**,**m**, The M2 (**k**) and M1-like (**m**) phenotypes of transfused CAR-Ms was assessed by flow cytometry and qPCR. Data shown are the mean ± s.e.m. (*n* = 4 per group). ***P < 0.001. Source data

**Extended Data Fig. 6:**
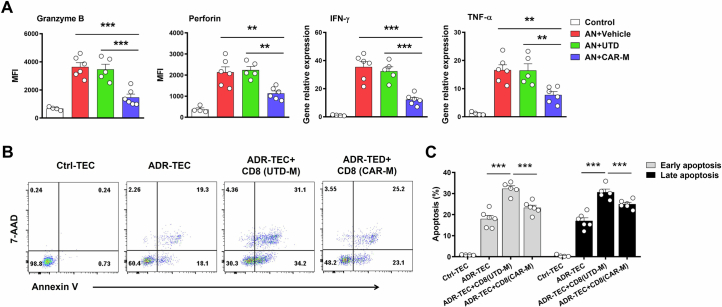
CAR-Ms suppressed CD8 T cell activation in AN mice and reduced cytotoxicity of CD8 T cells on TEC in vitro. (**A**) CD8 T cells were isolated from kidneys of AN mice treated with UTD-M or CAR-M. Inflammatory cytokines and cytotoxic molecules from CD8 T cells were assessed by flow cytometry and quantitative PCR. Data shown are the mean ± SEM (n = 4-6 per group). **P < 0.01, ***P < 0.001. (**B**, **C**) Tubular injury in AN mice is simulated by incubating kidney TECs with ADR for 24 hours. The injured kidney TECs (ADR-TECs) were then cocultured for 24 hours with CD8 T cells isolated from kidneys of AN mice treated with UTD-M or CAR-M. (**B**) Representative FACS analysis of apoptosis in TECs after 1-day coculture. (**C**) Frequency of early apoptosis (Annexin V + 7AAD– cells) and late apoptosis (Annexin V + 7AAD+ cells) in TECs after 1-day coculture. Data shown are the mean ± SEM (n = 4-6 per group). ***P < 0.001. Source data

To investigate the potential of CAR-M in a chronic kidney disease model (Adriamycin nephrosis (AN) in BALB/c mouse), we generated a new anti-TNF CAR construct including a transduction marker CD90.1 that facilitates sorting of CAR-expressing cells from BALB/c mice (Supplementary Fig. 7). Mice were treated with UTD-M, CARΔ-M, CAR-M or M2 at day 7 after Adriamycin (ADR) injection (Fig. 4a). Mice treated with CAR-M, but not UTD-M, CARΔ-M or M2, exhibited a significant improvement in kidney function, accompanied by a reduction in glomerulosclerosis, tubular damage and expression of inflammatory cytokines/chemokines in the kidney (Fig. 4b–g). It is worth noting that CAR-M-treated mice also demonstrated less kidney fibrosis, a result that could be explained by attenuation of early kidney inflammation and injury by CAR-Ms. We have previously demonstrated that the failed renoprotection of bone marrow M2 is due to the switch of transfused M2 macrophages from an anti-inflammatory to a pro-inflammatory phenotype in vivo^10^. Collectively, these data show the effectiveness of engineered CAR-Ms in treating experimental chronic kidney disease. The TNF level in inflamed kidney gradually increased from day 3 to day 14 and remained high until day 28 in AN mice. The TNF level in inflamed kidney of AN mice treated with CAR-Ms was markedly reduced at day 14 and day 28 in comparison with AN mice treated with UTD-Ms (Fig. 4h). Immunofluorescent co-staining for CD90.1 and CD206 in kidney sections demonstrated that most CD90.1^+^ CAR-Ms co-expressed M2 marker CD206 at day 28, but CARΔ-Ms did not (Fig. 4i). Infused CAR-Ms maintained an anti-inflammatory phenotype in response to persistently elevated TNF in inflamed kidney from day 14 to day 28 after ADR injection, but not in normal kidney (Fig. 4j,k). Moreover, infused CAR-Ms did not convert to a pro-inflammatory phenotype within inflamed kidney of AN mice at day 14 and day 28 (Fig. 4l,m). CD8 T cell is a key mediator of kidney injury in AN. To elucidate the protective mechanism of CAR-M against kidney injury in AN, we examined the phenotype and function of CD8 T cells isolated from the kidneys of CAR-M-treated AN mice. CD8 T cells obtained from the kidneys of AN mice or AN mice treated with UTD-Ms exhibited high levels of cytotoxic molecules and inflammatory cytokines. By contrast, the expression of these molecules was significantly reduced in CD8 T cells from AN mice treated with CAR-M. In addition, we performed a functional analysis of the cytotoxicity of CD8 T cells against kidney TECs in vitro. Apoptosis of TECs exposed to ADR was further increased by coculturing with CD8 T cells from AN mice treated with UTD-Ms, whereas it was reduced when cocultured with CD8 T cells from AN mice treated with CAR-M (Extended Data Fig. 6). Furthermore, CAR-M treatment reduced pro-inflammatory M1 endogenous macrophages but did not induce T_reg_ cells in the kidney of AN mice (Supplementary Fig. 8).

**Extended Data Fig. 7:**
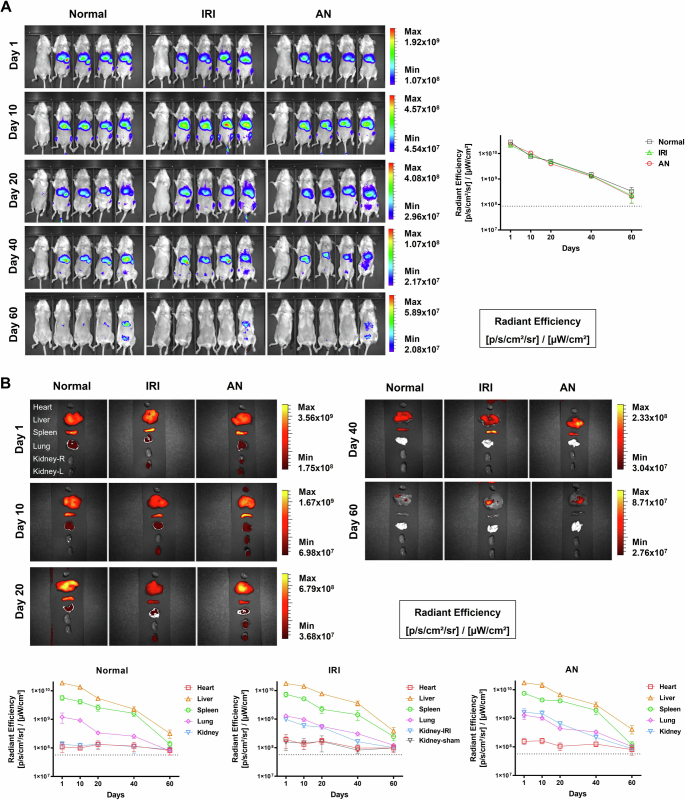
Tracking of CAR-M in vivo. (**A**) Biodistribution of XenoLight DiR-labeled CAR-Ms after IV administration in normal, renal IRI and AN mouse (n = 4 per group, the first mouse is a normal mouse that did not receive IV injection of CAR-Ms). Fluorescent signal was tracked over the course of 60 days (dotted line represents background fluorescence). The experiment was performed twice with similar results. (**B**) The normal, IRI and AN mice were euthanized, and organs were explanted for ex vivo imaging at 1, 10, 20, 40 and 60 days post injection (n = 4 mice per group). Macrophage mainly accumulated in liver, spleen, lung, and also infiltrated into diseased kidney. Source data

We next sought to evaluate the persistence and biodistribution of CAR-M after systemic administration under normal, acute and chronic inflammatory conditions. Fluorescently labelled CAR-Ms persisted at high level for at least 20 days within the body and then dramatically reduced from day 20 to day 60 in vivo. There was no difference among the three conditions (Extended Data Fig. 7a). To evaluate the ability of CAR-M to traffic to kidney and other organs, the relative signal in explanted whole-organ tissue was assessed at different time points in normal, IRI and AN mice. In all mice, fluorescently labelled CAR-Ms in liver, spleen and lung gradually decreased from day 1 to day 40 and dropped to near baseline at day 60. Fluorescently labelled CAR-Ms tended to distribute to inflamed kidney of IRI and AN mice and gradually decreased from day 1 to day 40, then dropped to near baseline at day 60 (Extended Data Fig. 7b).

### Infused CAR-Ms accumulated preferentially in liver and protected against liver IRI

**Fig. 5:**
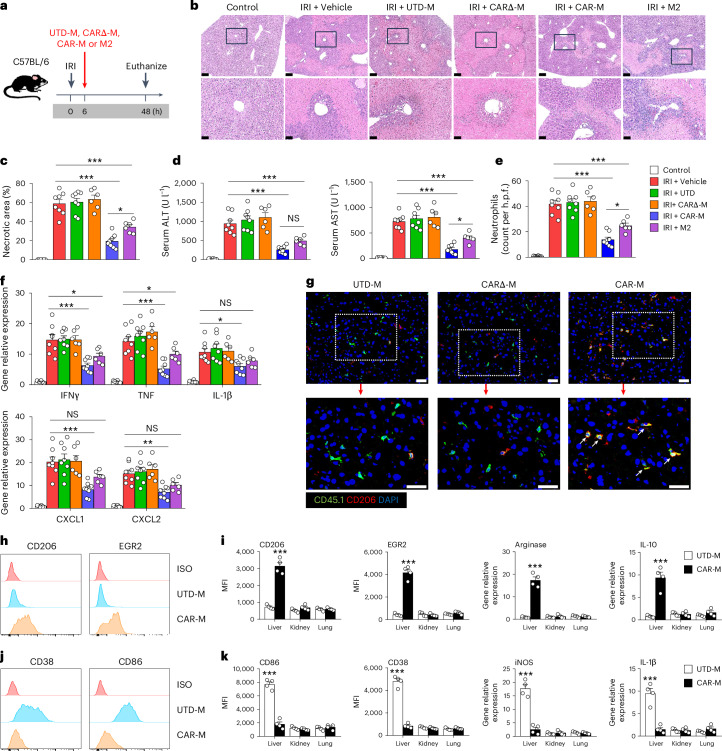
Evaluation of protective effect of CAR-M in acute liver injury model. **a**, C57BL/6 mice were treated with CD45.1^+^ UTD-M, CARΔ-M, CAR-M or M2 at 6 h after liver IRI. Mice were euthanized at 48 h after IRI. **b**, Representative haematoxylin and eosin-stained sections of liver from IRI mice treated with UTD-M, CARΔ-M, CAR-M or M2. Each panel shows low (top, bar = 250 µm) and high (bottom; corresponding to the boxed area, bar = 50 µm) power view. **c**, Quantitative assessment of necrotic areas in livers. *P < 0.05, ***P < 0.001. **d**, Serum alanine aminotransferase and aspartate aminotransferase levels were assessed in these mice. ALT, alanine aminotransferase; AST, aspartate aminotransferase. *P < 0.05, ***P < 0.001. **e**, Number of Gr-1^+^ neutrophils was assessed by immunofluorescence staining in liver. *P < 0.05, ***P < 0.001. **f**, mRNA levels of pro-inflammatory cytokines/chemokines in the livers. Data shown are the mean ± s.e.m. (*n* = 6–8 per group). *P < 0.05, **P < 0.01, ***P < 0.001. **g**, Immunofluorescence double staining of CD45.1 and CD206 in liver sections at 48 h after liver IRI. The white arrows indicate CD45.1^+^CD206^+^ CAR-Ms. Scale bars, 50 µm. **h**,**j**, CD45.1^+^ UTD-Ms and CD45.1^+^ CAR-Ms were sorted from various organs of mice at 48 h after liver IRI. Representative flow cytometric analysis of M2 markers (CD206 and EGR2, **h**) and M1 markers (CD38 and CD86, **j**) on transfused UTD-M and CAR-Ms from liver with IRI. **i**, The M2-like phenotype of transfused macrophages was assessed by flow cytometry (CD206 and EGR2) and qPCR (Arginase and IL-10). ***P < 0.001. **k**, The M1-like phenotype of transfused macrophages was assessed by flow cytometry (CD38 and CD86) and qPCR (iNOS and IL-1β). Data shown are the mean ± s.e.m. (*n* = 4 per group). ***P < 0.001. Source data

We next investigated the effect of CAR-Ms in liver IRI (Fig. 5a). Mice treated with CAR-M or M2 displayed a much milder liver injury compared to those treated with UTD-Ms or CARΔ-Ms evidenced by a significant decrease in areas of hepatocyte necrosis and lower serum levels of alanine aminotransferase and aspartate aminotransferase (Fig. 5b–d). Moreover, CAR-Ms and M2 treatment significantly reduced neutrophil infiltration and the production of pro-inflammatory cytokines/chemokines in the liver, including TNF, IL-1β, IL-6, CXCL1 and CXCL2 (Fig. 5e,f). However, CAR-Ms exhibited greater protective effect than M2 on hepatocyte necrosis, liver function and neutrophil infiltration in mice with liver IRI. Immunofluorescent co-staining for CD45.1 and CD206 in liver sections revealed that the majority of CD45.1^+^ CAR-Ms co-expressed the M2 marker CD206 at 48 h after liver IRI, but UTD-M and CARΔ-Ms did not (Fig. 5g). CAR-Ms, but not UTD-Ms, switched to anti-inflammatory phenotype in response to elevated TNF in inflamed liver, but not in kidney and lung at 48 h after liver IRI (Fig. 4h,i). It is worth noting that infused CAR-Ms did not convert to a pro-inflammatory phenotype within the inflamed liver (Fig. 4j,k). Furthermore, CAR-Ms treatment reduced pro-inflammatory M1 endogenous macrophages but did not induce T_reg_ cells in the liver of IRI mice (Supplementary Fig. 9). Together these findings suggest that engineered CAR-M hold potential as a cell therapy for inflammatory diseases in multiple organs.

### Generation and characterization of human CAR-Ms

**Fig. 6:**
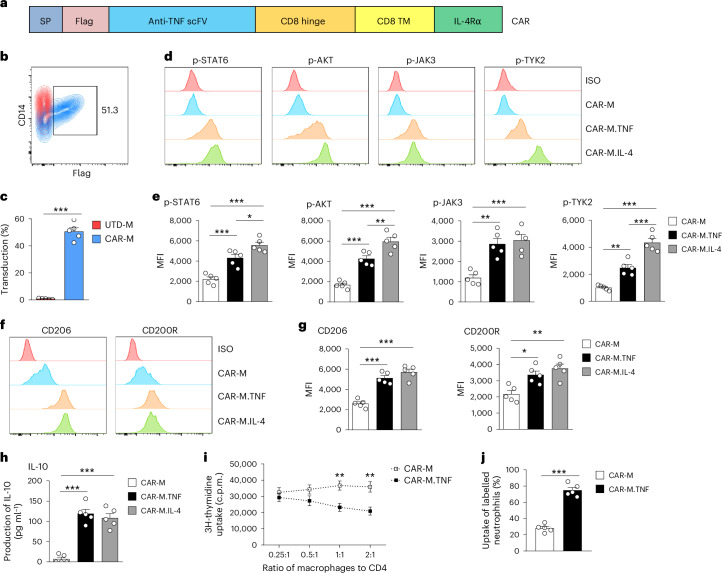
Generation and characterization of human CAR-Ms. **a**, Schematic representation of the anti-TNF CAR construct design for human macrophages. The CAR contains a Flag tag epitope, a scFv antibody specific for TNF, the hinge and transmembrane domains of human CD8α, and a human IL-4 receptor intracellular domain. **b**, Representative flow cytometric analysis of transduction efficiency (Flag-tag expression) on macrophages 3 days after transduction with CAR (blue, CAR-M; red, UTD-M). **c**, Quantitative analysis of the percentage of Flag+ cells on CAR-M and UTD-M. Data shown are the mean ± s.e.m. (*n* = 5 per group). ***P < 0.001. **d**, Representative flow cytometric analysis of IL-4 signalling pathways p-STAT6, p-AKT, p-JAK3 and p-TYK2 on CAR-M in response to TNF or IL-4. **e**, Flow cytometric analysis of mean fluorescence intensity (MFI) of IL-4 signalling pathways. Data shown are the mean ± s.e.m. (*n* = 5 per group) and are representative of two independent experiments. *P < 0.05, **P < 0.01, ***P < 0.001. **f**, Representative flow cytometric analysis of M2 markers CD206 and CD200R on CAR-M in response to TNF or IL-4. **g**, Flow cytometric analysis of MFI of M2 markers. Data shown are the mean ± s.e.m. (*n* = 5 per group) and are representative of 2 independent experiments. *P < 0.05; **P < 0.01; ***P < 0.001. **h**, Production of IL-10 by CAR-Ms after stimulation with TNF or IL-4. Data shown are the mean ± s.e.m. (*n* = 5 per group) and are representative of 2 independent experiments. ***P < 0.001. **i**, Inhibition of CD4 T-cell proliferation was examined with various dosages of CAR-Ms and CAR-M.TNF. Data shown are the mean ± s.e.m. (*n* = 5 per group) and are representative of 2 independent experiments. **P < 0.01 versus CAR-M. **j**, The human CAR-Ms and CAR-M.TNF were incubated with PKH26 Red-labelled apoptotic neutrophils for 3 h. The percentage of macrophages that had incorporated the apoptotic neutrophils was determined by flow cytometry. Data shown are the mean ± s.e.m. (*n* = 5 per group) and are representative of 2 independent experiments. ***P < 0.001. Source data

**Extended Data Fig. 8:**
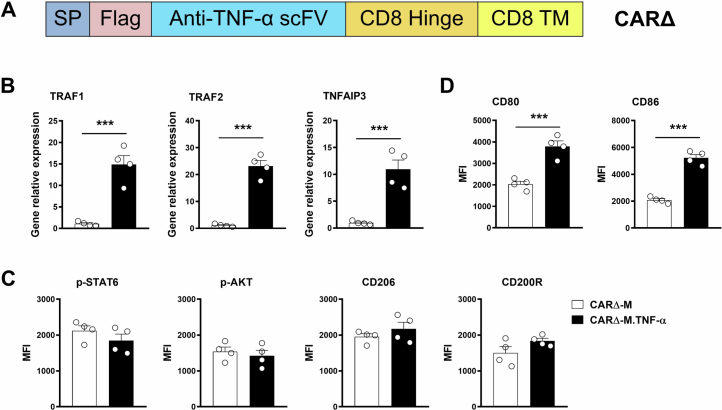
TNF-α did not induce M2 phenotype in human macrophages transduced with a truncated CAR (CARΔ). (**A**) CARΔ is a truncated CAR lacking the IL-4 receptor intracellular domain. (**B**) Quantitative PCR analysis of TNF-α signalling pathway on human CARΔ-M in response to TNF-α. (**C** and **D**) Flow cytometric analysis of M1 markers (CD80 and CD86) and M2 markers (p-STAT6, p-AKT, CD206 and CD200R) on human CARΔ-M in response to TNF-α. Data shown are the mean ± SEM (n = 4 per group) and are representative of 2 independent experiments. ***P < 0.001. Source data

To determine the translational potential of CAR-Ms, we generated a human anti-TNF CAR that contains an anti-human TNF scFv as the extracellular domain with the intracellular domain of human IL-4Rα (Fig. 6a). Lentivirus-transduced human macrophages expressed CAR efficiently (Fig. 6b,c). We next examined CAR-mediated redirection of macrophage phenotype in macrophages expressing intact CAR or CARΔ (Extended Data Fig. 8a). TNF stimulation selectively induced the IL-4 downstream signalling pathway as indicated by increased expression of p-STAT6 and p-AKT in CAR-Ms, whereas it exclusively triggered the TNF downstream signalling pathway in CARΔ-Ms (Fig. 6d,e and Extended Data Fig. 8b,c). Consistently, CAR-M.TNF but not CARΔ-M.TNF exhibited a significant upregulation of M2 markers, including CD206 and CD200R (Fig. 6f,g and Extended Data Fig. 8c). However, TNF stimulation of CARΔ-Ms resulted in upregulation of M1-associated markers: CD80 and CD86 (Extended Data Fig. 8d). Compared with IL-4 stimulation, TNF preferably activated p-JAK3 (IL-2Rγc chain), but not p-TYK2 (IL-13Rα1 chain) on CAR-Ms, indicating TNF mainly activated type 1 IL-4 receptor complex through CAR on human macrophages (Fig. 6d,e). CAR-Ms produced equivalent levels of IL-10 in response to TNF or IL-4 (Fig. 6h), suggesting their anti-inflammatory capability. Compared to CAR-Ms, CAR-M.TNF significantly suppressed T-cell proliferation in vitro (Fig. 6i). CAR-M.TNF enhanced the clearance of dead cells compared to CAR-Ms, suggesting an augmented wound-healing capacity (Fig. 6j). These findings demonstrate that anti-TNF CAR redirects human macrophage responses away from endogenous TNF signalling towards immunosuppressive and tissue-repair pathways.

## Discussion

**Fig. 7:**
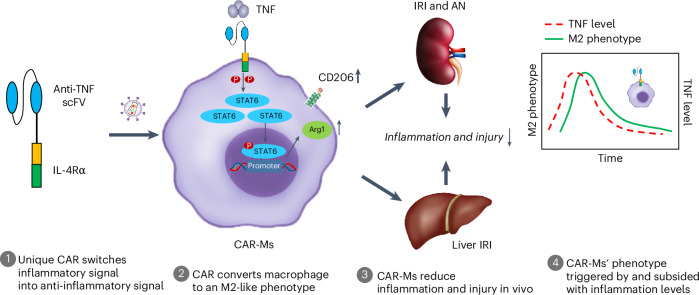
CAR-Ms as inflammatory disease immunotherapy. Our engineered CAR structure integrates an anti-TNF scFv as the extracellular domain with the intracellular domain of IL-4 receptor alpha (IL-4Rα). This design enables the capture of TNF and converts its signal into an IL-4 signal, promoting an anti-inflammatory response on macrophages. When capturing TNF, CAR-Ms exhibit an M2-like phenotype including elevated p-STAT6 expression and increased M2 markers (CD206, Arginase 1). In vivo, CAR-Ms reduce inflammation and injury in acute and chronic kidney inflammatory diseases (renal IRI and AN models), demonstrating substantial therapeutic potential. Remarkably, the CAR-Ms switched to the M2 phenotype only at the site of inflammation and reverted once the inflammation subsided, ensuring targeted and timely anti-inflammatory responses. CAR-Ms also protect against liver IRI, suggesting potential applications for inflammatory diseases in multiple organs.

Inflammation is a key driver of numerous diseases including kidney and liver diseases, arthritis, obesity, diabetes, cardiovascular diseases, neurological disorders and cancer. Failure to resolve inflammation is a pivotal pathological mechanism that drives disease progression^23^. Currently available drugs for treating inflammation, particularly in chronic inflammatory diseases, are toxic and limited in their efficacy^1^. Administration of M2-like macrophages has demonstrated effectiveness in resolving inflammation and reducing tissue injury^5–7,9,24^. However, a substantial challenge to macrophage therapy is their plasticity, which allows them to alter their phenotype in response to inflammatory microenvironments in a diseased organ, sometimes reverting to an inflammatory M1 phenotype^10,12,25^. By transforming an inflammatory stimulus into a driver of wound healing and inflammatory resolution, CAR technology offers a promising approach to harness and stabilize the phenotype of macrophages effectively (Fig. 7).

We proposed that macrophages equipped with a CAR could exploit pro-inflammatory mediators in an inflamed organ to switch their phenotype to anti-inflammatory, thereby reducing tissue injury. Our CAR approach is distinguished by its ability to direct cell phenotype and function to suppress immune responses, whereas other CARs are used to enhance existing cell functions and boost immune responses. This CAR design allowed the anti-TNF scFv to specifically bind TNF at the site of inflammation, triggering a signal switch towards an anti-inflammatory response via its connection to the IL-4ra domain. When stimulated by TNF, CAR-Ms showed a notable transformation in phenotype towards that of M2 macrophages, as evidenced by upregulation of M2-associated genes and functional pathways related to tissue remodelling and efferocytosis. The strategy of converting one type of signal into another has been employed previously to improve the efficiency of CAR T-cell therapy for cancer. For instance, Vera and colleagues improved CAR T-cell function by introducing an IL-4/IL-7 invert cytokine receptor (ICR) to counteract the immunosuppressive tumour microenvironment in pancreatic cancer^26^. Similarly, Gottschalk and colleagues used a granulocyte-macrophage colony-stimulating factor (GM-CSF)/IL-18 ICR to sustain the functionality of CAR T cells targeting human epidermal growth factor receptor 2, a metastatic colorectal cancer target^27^. These studies successfully transformed the signals generated by GM-CSF and IL-4 into signals associated with IL-18 and IL-7, respectively. While the ICR serves as an enhancer to improve the anti-tumour activity of CAR T-cell therapy, our anti-TNF CAR has the unique capability of redirecting macrophage phenotype and function, thereby reversing inflammation in acute and chronic diseases. In alignment with the ICR approach in cancer therapy, our CAR-mediated signal switch presents a novel strategy to reshape immune response across a variety of diseases.

This CAR-M approach exhibits unique features that differentiate it from other CAR-engineered immune cell therapies. Unlike CAR T-cell therapy, with its risk of cytokine release syndrome, macrophages lack the ability to self-proliferate, and their limited numbers mitigate this risk^28^. Our CAR-Ms exhibited robust functionality as long as inflammation persisted; they maintained an M2-like phenotype for several days during acute kidney injury and for several weeks in chronic kidney inflammation. It is worth noting that the M2-like phenotype of CAR-Ms faded as inflammation resolved in acute IRI, highlighting that the functionality of CAR-Ms is contingent upon the intensity of the inflammatory milieu. It is worth noting that our CAR-Ms are immunosuppressive, a departure from most CAR T-cell therapies designed for cell deletion, such as targeting cancer or autoimmune cells^19,29,30^. CAR-Ms exhibit distinctive reparative functions, including the clearance of damaged cells and the promotion of tissue regeneration, whereas CAR T_reg_ cells mainly possess immune suppression capabilities^31,32^. Our CAR-Ms exhibited precise targeting to the site of inflammation. CAR-Ms had an induced anti-inflammatory phenotype exclusively in the inflamed organ, but not in other organs where macrophages typically accumulate, such as the liver, spleen and lungs^18^. This indicates that the functionality of CAR-Ms is specific to inflammation. In general, when cells are genetically engineered with specific genes, these gene functions typically remain activated unless specific switch systems are integrated^33^. One side effect arising from sustained on-target recognition, as seen in the case of CAR T cells targeting CD19, is the prolonged impact on healthy B cells^34,35^. By contrast, our CAR-Ms were switched off once inflammation subsided. Moreover, Our CAR-Ms remain in the body for weeks, which is much shorter than the years CAR T cells can persist, indicating that macrophage therapy poses fewer safety risks. Furthermore, our CAR-Ms are triggered by soluble inflammatory cytokines, while most of CAR T cells are activated by surface antigens. We demonstrated that CAR-M can induce an immune response triggered by both soluble and non-soluble TNF.

In this study, the ability of CAR-M to reduce inflammation in various organ models of both acute and chronic inflammation has been demonstrated, although notable challenges remain for their clinical application. The engineered human macrophages with human CAR structures exhibit similar characteristics to mouse CAR-M in vitro, showing that the anti-TNF CAR redirects human macrophage responses away from endogenous TNF signalling towards anti-inflammatory and tissue-repair pathways. This crucial evidence lays the groundwork for validating the potential of CAR-M through rigorous testing in additional preclinical models, paving the way for future clinical trials in human inflammatory diseases. Ongoing clinical trials in various diseases are exploring the potential therapeutic application of ex vivo generated M2 macrophages^13,14^. Nevertheless, there is a concern that their phenotypes may revert in the face of pathogenic conditions. The development of our novel CAR-M has addressed this inherent drawback, offering a potential solution for the future application of macrophage-based cell therapy across a range of human diseases.

In conclusion, this study has demonstrated CAR-mediated signal switching as a successful approach for altering macrophage function to effectively attenuate both acute and chronic inflammation. The anti-inflammatory actions of CAR-M are triggered by inflammatory cytokines, vary in parallel with levels of inflammation and deactivate following resolution of inflammation. This innovative approach may be applicable for the development of cell therapies against a wide range of diseases.

## Methods

### CAR construction and lentivirus production

The anti-TNF scFv was constructed using the variable regions of the heavy and light chains (VH and VL, respectively) of TNF-binding antibody (clone, MP6-XT22) and synthesizing gene fragments with the VH-(G4S)3-VL architecture (Vectorbuilder). The first anti-TNF CAR contains a Flag tag epitope, an anti-TNF scFv, the hinge and transmembrane domains of mouse CD8α, and a mouse IL-4 receptor intracellular domain. CARΔ is a truncated CAR lacking the IL-4 receptor intracellular domain. The second anti-TNF CAR was connected via a P2A ‘self-cleaving’ peptide to CD90.1, a marker that indicates transduction efficiency and also facilitates sorting of CAR-expressing cells. Human anti-TNF CAR construct comprises an anti-TNF scFv (Infliximab), the hinge and transmembrane domains of human CD8α, and the intracellular domain of the human IL-4 receptor alpha. Complementary DNAs were synthesized and cloned into the third-generation lentivirus backbone by Vectorbuilder. Lentiviral production was performed according to a protocol used by Children’s Medical Research Institute. In brief, Lenti-X 293 T cells were at 80% confluency and cotransfected with anti-TNF CAR plasmids and the lentiviral packaging plasmids (pMDLg/pRRE, pRSV/REV and PMD2.G from Addgene). The media on the Lenti-X 293 T cells was replaced with fresh media 8 h after transfection. The supernatant was collected at 24–72 h after transfection. The virus was concentrated by centrifugation at 40,000 × *g* for 4 h.

### Mouse bone marrow macrophage differentiation and transduction

BMDMs were produced as previously described^10,36^. Differentiation was confirmed by F4/80 staining on day 6. The BMDMs were cultured for 24 h with LPS (100 ng ml^−1^) and IFNγ (20 ng ml^−1^) to become M1 macrophages, with IL-4/13 (10 ng ml^−1^ each) to become M2 macrophages. The BMDMs were transduced with concentrated lentivirus after 4 days of differentiation, then cultured for another 2 days with M-CSF (20 ng ml^−1^). A multiplicity of infection of 500 infectious units per cell was used unless otherwise stated.

### Human macrophage differentiation and transduction

Peripheral blood mononuclear cells (PBMCs) were isolated from human blood samples. All samples were obtained with informed patient consent and in accordance with local ethics approval from the Human Research Ethics Committee of Western Sydney Local Health District. The blood monocytes/macrophages were enriched by CD14^+^ MicroBeads (Miltenyi Biotec, Germany) and further purified by cell attachment for 4 h. Blood monocytes/macrophages were cultured in RPMI 1640 medium, supplemented with 10% fetal bovine serum, penicillin (100 U ml^−1^) and streptomycin (100 µg ml^−1^) plus 50 ng ml^−1^ M-CSF for 6 days. The purity of macrophages was above 95% as estimated by fluorescence-activated cell sorting (FACS) analysis. The human macrophages were transduced with concentrated lentivirus after 4 days of differentiation, then cultured for another 2 days with M-CSF. A multiplicity of infection of 500 infectious units per cell was used unless otherwise stated.

### CAR-Ms culture with soluble and immobilized TNF

Briefly, TNF was immobilized in cell culture wells by covering each well with 100 ng ml^−1^ TNF at room temperature for 30 min, washing once with PBS, then allowing the well to dry. CAR-Ms were exposed to medium alone, 10 ng ml^−1^ soluble TNF or immobilized TNF for 24 h. The phenotype of CAR-Ms was assessed by flow cytometry and quantitative PCR (qPCR).

### In vitro suppression assay

CD4^+^ T cells isolated from splenocytes or PBMCs (1 × 10^5^ cells per well) were added into 96-well plates in the presence of pre-adherent mouse or human CAR-M and stimulated by anti-mouse or human CD3/CD28 (100 ng ml^−1^ and 200 ng ml^−1^) and 50 U ml^−1^ of mouse or human IL-2 for 48 h and then [3H]thymidine (1 microcuries per well) was added and the incubation continued for a further 16 h. Cells were collected using a Packard Filtermate Harvester 96 and counted by Microbeta counter (PerkinElmer).

### CAR-Ms coculture with M1

Co-culture of two monolayers of cells was as described previously^5,37^. CAR-Ms were grown on 25-mm-diameter plastic coverslips (Nunc) and stimulated with TNF (10 ng ml^−1^) to become TNF-activated CAR-M. Macrophages also were seeded onto 6-well plates and further processed to become M1 macrophages. The coverslips seeded with monolayers of CAR-Ms were placed over confluent monolayers of M1. The phenotype of M1 was assessed after 24 h co-culture.

### Phagocytic activity

The UTD-Ms and CAR-Ms were cocultured with 1 mg ml^−1^ FITC-labelled dextran (40,000 kDa; Molecular Probes) in the presence or absence of TNF for 45 min at 37 °C. To determine phagocytic activity, the uptake of FITC-labelled dextran was detected by flow cytometry. Neutrophils were isolated from bone marrow cells or PBMCs by flow cytometry as described previously^38^. Isolated neutrophils were exposed to UV irradiation for 15 min to induce apoptosis and then incubated at 37 °C for 4 h. The UTD-Ms and TNF-activated CAR-Ms were incubated with PKH26 Red-labelled apoptotic cells for 3 h. The uptake of apoptotic neutrophils was determined by flow cytometry.

### Animal studies

BALB/c, C57BL/6 (CD45.2^+^), congenic C57BL/6 (CD45.1^+^) and TNF-deficient (B6⋅129S-Tnftm1Gkl/J) mice were purchased from the Australian BioResources. For all studies, adult (8–12 weeks of age) male mice were used in accordance with the animal care and use protocol approved by the Animal Ethics Committee of Western Sydney Local Health District.

Kidney IRI was produced in C57BL/6 mice as previously described^39,40^. Briefly, via a midline abdominal incision, ischaemia was induced in both kidneys for 30 min at 37.0 °C or only the left kidney for 30 min at 37 °C (unilateral IRI). Animals subjected to sham operation were used as controls. For CAR-M treatment, 4 × 10^6^ CAR-Ms or UTD-Ms were transferred into C57BL/6 or TNF-deficient mice by a single tail-vein injection at 6 h after ischaemia. Mice were euthanized at indicated time points after reperfusion to collect blood and tissues for further analysis. The biodistribution of XenoLight DiR-labelled UTD-M or CAR-M was assessed in the unilateral IRI model. The heart, liver, lung, spleen and kidneys were collected at various time points for ex vivo imaging using an IVIS Spectrum (PerkinElmer).

AN is a model of chronic kidney disease due to focal segmental glomerulonephritis and was induced as previously described^36^. Dose-finding studies defined an optimal dose of ADR at 9.6 mg kg^−1^ body weight (Pharmacia & Upjohn) for BALB/c mice. ADR was injected once via the tail vein of each mouse. BALB/c mice were treated with 4 × 10^6^ CAR-Ms or UTD-Ms by a single tail-vein injection at day 7 after ADR injection. Mice were euthanized at indicated time points after ADR injection to collect blood, urine and tissues for further analysis. The biodistribution of XenoLight DiR-labelled CAR-M was assessed in the AN model. The kidney and other organs were collected at various time points for ex vivo imaging.

Partial liver ischaemia was induced as previously described^41^. Briefly, fasted mice were anesthetized with a ketamine/xylazine mixture. After a midline laparotomy, an atraumatic clip (Roboz) was placed across the portal vein and hepatic artery to interrupt blood supply to the left lateral/median lobes (70%) of the liver for 60 min at 37.0 °C. Sham-operated mice underwent the same surgical procedure without vascular occlusion. For CAR-M treatment, 2 × 10^6^ CAR-Ms or UTD-Ms were transferred into C57BL/6 mice by a single tail-vein injection at 6 h after ischaemia. All mice were euthanized at 48 h after IRI.

### Cell suspensions preparation

Spleen was isolated, minced and digested for 30 min at 37°C in RPMI 1640 containing 1 mg ml^−1^ collagenase D (Roche) and 100 μg ml^−1^ DNase I (Roche). The digested cell suspension was then passed through a 40 μm cell strainer. Kidney, liver and lung were perfused with saline before removal and digested with collagenase and DNase as previously described^42^. Kidney, liver and lung were cut into 1 to 2 mm^3^ pieces and placed in DMEM containing 1 mg ml^−1^ collagenase IV (Sigma Aldrich) and 100 μg ml^−1^ DNase I (Roche) for 40 min at 37°C with intermittent agitation. The digested cell suspension was then passed through a 40 μm cell strainer. The transfused CAR-Ms or UTD-Ms were sorted from various organs by FACS for further analysis.

### Primary culture of mouse renal TECs

Primary mouse TECs were generated following established methods adapted from Doctor et al.^43^. In brief, the kidneys were collected after cardiac perfusion with saline to remove blood cells. The tissue from the outer cortex was cut into pieces of approximately 1 mm^3^ and then digested in DMEM containing 1 mg ml^−1^ collagenase IV (Sigma Aldrich) and 100 μg ml^−1^ DNase I (Roche) for 40 min at 37 °C with intermittent agitation. Kidney TECs were separated by centrifugation using Percoll solution and cultured in defined K1 medium: DMEM/F12 medium supplemented with 10 ng ml^−1^ epidermal growth factor, 1 ng ml^−1^ prostaglandin E_1_, 5 µg ml^−1^ insulin, 5 µg ml^−1^ transferrin, 5 ng ml^−1^ sodium selenite, 5 × 10^–11^ M triiodothyronine, 5 × 10^–8^ M hydrocortisone, 100 U ml^−1^ penicillin, 100 µg ml^−1^ streptomycin, 25 mM HEPES and 5% fetal bovine serum.

### Simulated ischaemic kidney TECs coculture with CAR-Ms

Ischaemia in kidney TECs was simulated by immersing the cellular monolayer in mineral oil according to the protocol of Meldrum et al.^44^. This immersion simulated ischaemia by restricting cellular exposure to oxygen and nutrients as well as by limiting metabolite washout. Briefly, kidney TECs (1 × 10^5^) were placed in 6-well tissue culture plates in serum-free K1 medium for 24 h, washed twice with PBS and immersed in mineral oil for 60 min at 37 °C. After washing with PBS, TECs were incubated in K1 medium. CAR-Ms were grown on 25-mm-diameter plastic coverslips (Nunc) and stimulated with TNF (10 ng ml^−1^) to become TNF-activated CAR-Ms. The coverslips seeded with monolayers of TNF-activated CAR-M or UTD-Ms were placed over confluent monolayers of TEC. TECs were exposed to serum-free K1 medium alone as the non-ischaemic control. Apoptosis of TECs at 1 day after the coculture was measured by staining with 7-aminoactinomycin D (7-AAD) and annexin V following the manufacturer’s protocol (BD Biosciences). The number of kidney TECs was determined at each time point by trypsinizing and counting the cells.

### Kidney TECs coculture with CD8 T cells

CD8 T cells were isolated by flow cytometry from kidneys of AN mice treated with UTD-Ms or CAR-Ms at day 28 after ADR injection. Tubular cell injury in AN mice was simulated by incubation with ADR in vitro. Briefly, kidney TECs (1 × 10^5^) were incubated in serum-free K1 medium with ADR (1 mM) in 6-well tissue culture plates for 24 h. After extensive washing with PBS, TECs (1 × 10^5^) were cocultured with FACS-sorted CD8 T cells (2 × 10^5^) for 24 h. TECs were exposed to serum-free K1 medium alone as a control. Apoptosis of TECs at 1 day after the coculture was measured by staining with 7-AAD and annexin V following the manufacturer’s protocol (BD Biosciences).

### Flow cytometry and cell sorting

To analyse the purity of cultured mouse macrophages, cells were stained with Fc block/anti-CD16/32 (2.4G2) and antibodies to CD11b (M1/70) and F4/80 (BM8). Transduction efficiency of the CAR-Ms was tested by staining with antibodies to F4/80 (BM8), Flag-tag (L5) and CD90.1 (HIS51). M1 and M2 markers on primary macrophages were detected with antibodies against pSTAT6 (CHI2S4N), pAKT (J1-223.371), pJAK3 (JAK3Y980981-E10), pTYK2, CD206 (C068C2), EGR2 (Erongr2), PD-L2 (TY25), CD86 (GL1) and CD38 (90). When FACS sorting was performed on the digested organs, single-cell suspensions were pregated on haematopoietic cells using anti-CD45 antibody (30-F11), then 7-AAD was used to exclude dead cells. Transfused CD45.1^+^ UTD-Ms, CD45.1^+^ CAR-Ms and CD90.1^+^ CAR-Ms were sorted using a FACSAria II (BD). After sorting, cells were used for phenotypic and functional assays. Other antibodies used in this study included CD3 (145-2C11), CD4 (GK1.5), CD8a (53-6.7), Granzyme B (NGZB), Perforin (eBioOMAK-D) and Foxp3 (FJK-16s) as well as corresponding isotype controls, all purchased from eBioscience or Biolegend.

For FACS analysis of human macrophages, single-cell suspensions were stained with antibodies to CD14 (M5E2), pSTAT6 (CHI2S4N), pAKT (J1-223.371), pJAK3 (JAK3Y980981-E10), pTYK2, CD80 (2D10.4), CD86 (IT2), CD206 (19.2) and CD200R (OX108) to assess macrophage phenotypes.

### RNA sequencing of macrophages

RNA was isolated from mouse macrophages and treated as described in each figure using Ambion RiboPure RNA Purification Kit (AM1924, Thermo Fisher). Poly-A selected messenger RNA libraries were generated using the Illumina stranded mRNA Prep Kit (20040534, Illumina) and validated with the Agilent Tapestation 4200 and qPCR before sequencing. Short-read sequencing was performed on the Novaseq X platform (Illumina) with 150 bp pair-end reads. Library sequencing quality was determined using FastQC (Babraham Bioinformatics: www.bioinformatics.babraham.ac.uk/). Illumina adaptor sequence and low quality read trimming (read pair removed if <20 base pairs) was performed using Trim Galore (Babraham Bioinformatics). STAR (2.7.8a) was used to align reads to mouse genome mm10 using ENSEMBL gene annotations as a guide^45^. Read counts data corresponding to ENSEMBL gene annotations were generated with the STAR flag–quantMode GeneCounts. Multiqc (1.13) was used to verify quality metrics. All analyses were performed in the R Statistical Environment (4.3.2) with tidyverse. Briefly, counts data were background corrected and normalized for library size using edgeR (4.1.29). Differential gene expression (DGE) was determined using the quasi-likelihood F-test (QLFtest) (Benjamini–Hochberg multiple testing correction *P* < 0.05). Gene lists were functionally annotated with Gene Ontology, Reactome and Kyoto Encyclopedia of Genes and Genomes (KEGG) pathways (adjusted *P* < 0.05) using the cluster Profiler package (4.11.0).

### qPCR

Total RNA was isolated from tissue or cells by RNeasy Mini Kit (Qiagen), then reverse-transcribed into cDNA with First Strand cDNA Synthesis Kit (Invitrogen). Real-time PCR was performed on the CFX96 Touch Real-Time PCR Detection System (Bio-Rad) using the SYBR mastermix (Invitrogen). The data were normalized to housekeeping gene expression and quantified using the 2-ΔΔCt method. The primer sequences of the target genes are shown in Supplementary Table 1.

### ELISA of cytokines

IL-1β, IL-6, IL-10 and TNF levels in culture supernatants or serum were assayed using an enzyme-linked immunosorbent assay (ELISA) kit (Thermo Fisher). ELISA was performed according to the manufacturer’s protocol.

### Terminal deoxynucleotidyl transferase–mediated dUTP nick end-labelling assay

Apoptotic cells in the IRI kidney were detected by terminal deoxynucleotidyl transferase–mediated 2′-deoxyuridine 5′-triphosphate (dUTP) nick end-labelling assay following the manufacturer’s protocol (Roche Diagnostics). Briefly, the fixed frozen sections were permeabilized with freshly prepared 0.1% Triton X-100 in 0.1% sodium citrate for 5 min on ice. DNA fragments in apoptotic cells were then labelled and identified by terminal transferase dUTP conjugated with tetramethylrhodamine red for 60 min at 37 °C. The number of apoptotic cells was counted in 8 to 10 non-overlapping fields in the corticomedullary junction and outer medulla in a blinded manner.

### Histology and immunofluorescence

Kidney sections were stained with periodic acid–Schiff (PAS). Tubular injury score of kidney IRI in the corticomedullary junction and outer medulla were evaluated semiquantitatively. Briefly, tubular damage was estimated in 8–12 high-power fields per section by using a scoring system on the basis of the percentage of damaged tubules per field (0, normal; 1, 10%; 2, 11–25%; 3, 26–50%; 4, 51–75%; and 5, 75%). The mean score of each animal was compared. Glomerulosclerosis, tubular damage and kidney fibrosis in AN mice were evaluated using previously described methods^46^. The degree of glomerulosclerosis was measured using a quantitative method. The outline of the glomerular capillary tuft was traced, and the computed area was used as a measure of total glomerular area. The area covered by PAS-positive staining in the same glomerulus was then determined. The percentage of glomerulosclerosis for each glomerulus was calculated by dividing the total PAS-positive area by the total glomerular area. The mean value of 20 randomly selected glomeruli was determined for each section. Damaged tubules were identified by the presence of diffuse tubular dilation, intraluminal casts and/or tubular cell vacuolization, and detachment in cortex and medulla in 8–12 high-power fields (×200 magnification) per PAS-stained section, in a blinded fashion. The number of damaged tubules was divided by the number of the total tubules in the same field to obtain the percentage of damaged tubules. Kidney sections were stained for fibrosis using Gomori trichrome. Quantitation for Gomori trichrome staining was performed by a modified grid-counting method in 8–12 high-power fields (×200 magnification) per section, in a blinded fashion. Liver sections were stained with haematoxylin and eosin for examination of necrotic areas. All slides were blindly quantified in 10–12 high-power fields where the percentage of necrosis was calculated from the total area of the tissue section. The data were then averaged to calculate the necrotic area for each mouse. For lung injury, vascular congestion, oedema and inflammatory cell infiltration were assessed, and for liver injury, hepatocellular necrosis, portal inflammation and inflammatory cell infiltration were evaluated. Each parameter was graded on a scale of 0–3 (none, 0; mild, 1; moderate, 2; and severe, 3). The total pathology scores for the lungs and livers were expressed as the sum of the score for all parameters^47^. The spleen injury scoring system was graded on a scale of 0–4 (none, 0; minimal, 1; mild, 2; moderate, 3; and severe, 4). Pathological scoring of the heart was graded with a scale of 0–4, where 0 was without inflammation; 1 was inflammatory mononuclear foci ≤5% area; 2 was inflammatory mononuclear foci >5% but not over 20% area; 3 was inflammatory mononuclear foci >20% area but without necrosis; 4 was diffuse inflammation with necrosis^48^. To avoid selection bias, the areas to be viewed for morphometric analysis were anatomically identical for each section and positioned before microscopic visualization.

For immunofluorescence staining of CAR-M, frozen sections were stained with mouse anti-mouse CD45.1 (A20, 1/100, Biolegend), mouse anti-mouse anti-CD90.1 (HIS51, 1/200, eBiosciences) and rat anti-mouse CD206 (C068C2, 1/50, Biolegend) and then incubated with the secondary antibodies, AF488 goat anti-mouse IgG (1/1,000; Invitrogen) and AF546 goat anti-rat IgG (1/1,000; Invitrogen). For immunofluorescence staining of neutrophils and proliferated tubular cells, rat anti-mouse Gr-1 (Ly-6G, 1/100; Biolegend) and rat anti-mouse Ki67 (11F6, 1/40; Biolegend) were used as the primary antibody and AF488 or AF546 goat anti-rat IgG as the secondary antibody. Control rat or mouse IgG to primary antibodies was included in staining. CD45.1^+^CD206^+^ CAR-Ms and CD90.1^+^CD206^+^ CAR-Ms were identified in kidney and liver sections. The number of interstitial Gr-1-positive cells was quantitated in 8–10 non-overlapping fields of kidney and liver sections.

### Statistics

Statistical tests included unpaired, two-tailed Student’s *t*-test using Welch’s correction for unequal variances and one-way or two-way analysis of variance with Tukey’s or Sidak’s multiple comparison test. Statistical analyses were performed using Prism (version 8, GraphPad). Results are expressed as the mean ± s.e.m. *P* < 0.05 was considered statistically significant.

### Reporting summary

Further information on research design is available in the Nature Portfolio Reporting Summary linked to this article.

## Supplementary information

Supplementary Information Supplementary Figs. 1–9, Table 1 and nucleotide sequences of CAR constructs.

Reporting Summary

## Source data

Source Data Fig. 1 Statistical source data.

Source Data Fig. 2 Statistical source data.

Source Data Fig. 3 Statistical source data.

Source Data Fig. 4 Statistical source data.

Source Data Fig. 5 Statistical source data.

Source Data Fig. 6 Statistical source data.

Source Data Extended Data Figs. 1–8 Statistical source data.

## Data Availability

The main data supporting the results in the study are available within the paper and its Supplementary Information. RNA sequencing data that support the findings of this study will be deposited and made publicly available in the NCBI Gene Expression Omnibus repository under accession number GSE250551. Source data are provided with this paper.
