# Supplementary material for: Targeting inflammation with chimeric antigen receptor macrophages using a signal switch
Source: Nat Biomed Eng. 2025 May 7;9(9):1502–16. doi: 10.1038/s41551-025-01387-8 (PMC12443588; doi:10.1038/s41551-025-01387-8)
Supplement: Supplementary file 1 — Supplementary Figs. 1–9, Table 1 and nucleotide sequences of CAR constructs. [file 41551_2025_1387_MOESM1_ESM.pdf]

nature biomedical engineering

Supplementary information https://doi.org/10.1038/s41551-025-01387-8

Targeting inflammation with chimeric antigen receptor macrophages using a signal switch

In the format provided by the authors and unedited

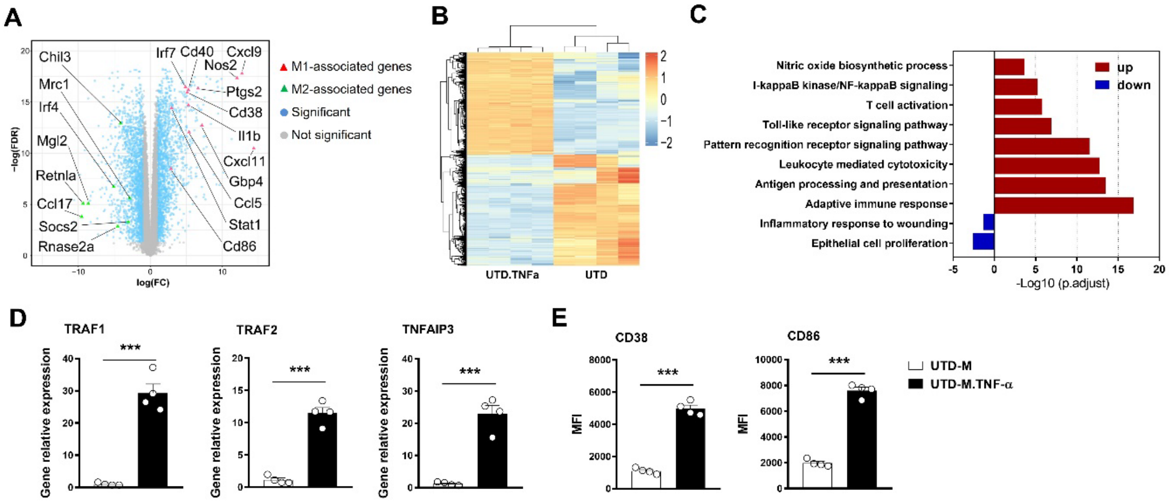

Supplementary Figure 1. TNF-α induced M1 phenotype on UTD macrophages.

(A) Volcano plot of differentially expressed genes in UTD-M.TNF-α versus UTD-M. Blue indicates Padj < 0.05 and log2 fold change >1 or <−1. Red triangles indicate significant M1-associated genes and green triangles indicate significant M2-associated genes (n = 4 per group). Statistical significance was calculated using the QLFtest for DGE data. (B) Hierarchical clustering of differentially expressed genes from UTD-M.TNF-α versus UTD-M (n=4 per group). The heatmap shows log2 fold-change in gene expression relative to UTD-M. (C) GO and KEGG pathway analysis results of up- and down-regulated pathways in UTD-M.TNF-α, compared to UTD-M. (D) Quantitative PCR analysis of TNF-α signalling pathway on UTD-M in response to TNF-α. (E) Flow cytometric analysis of mouse M1 markers (CD38 and CD86) on UTD-M in response to TNF-α. Data shown are the mean ± SEM (n=4 per group) and are representative of 2 independent experiments. ***P<0.001.

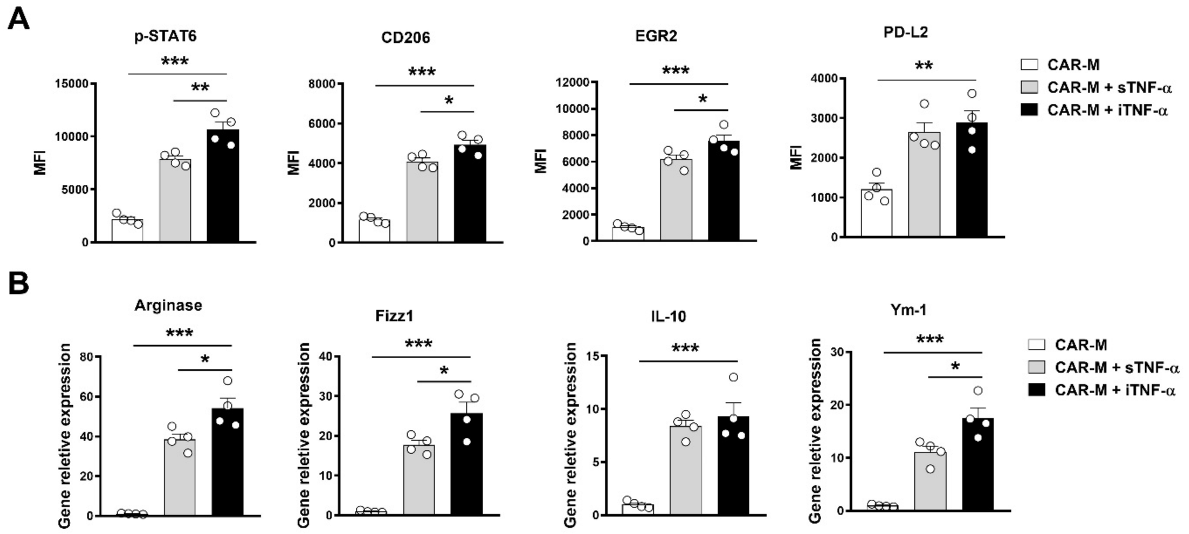

Supplementary Figure 2. Soluble and immobilized TNF-α induced M2-like phenotype on CAR-Ms.

(A. B) CAR-Ms were exposed to medium alone, 10 ng/mL soluble (s) TNF-α or immobilized (i) TNF-α for 24 hours. The M2-like phenotype of CAR-Ms was determined by flow cytometry (A) and quantitative PCR (B). Data shown are the mean ± SEM (n=4 per group) and are representative of 2 independent experiments. *P<0.05, **P<0.01, ***P<0.001.

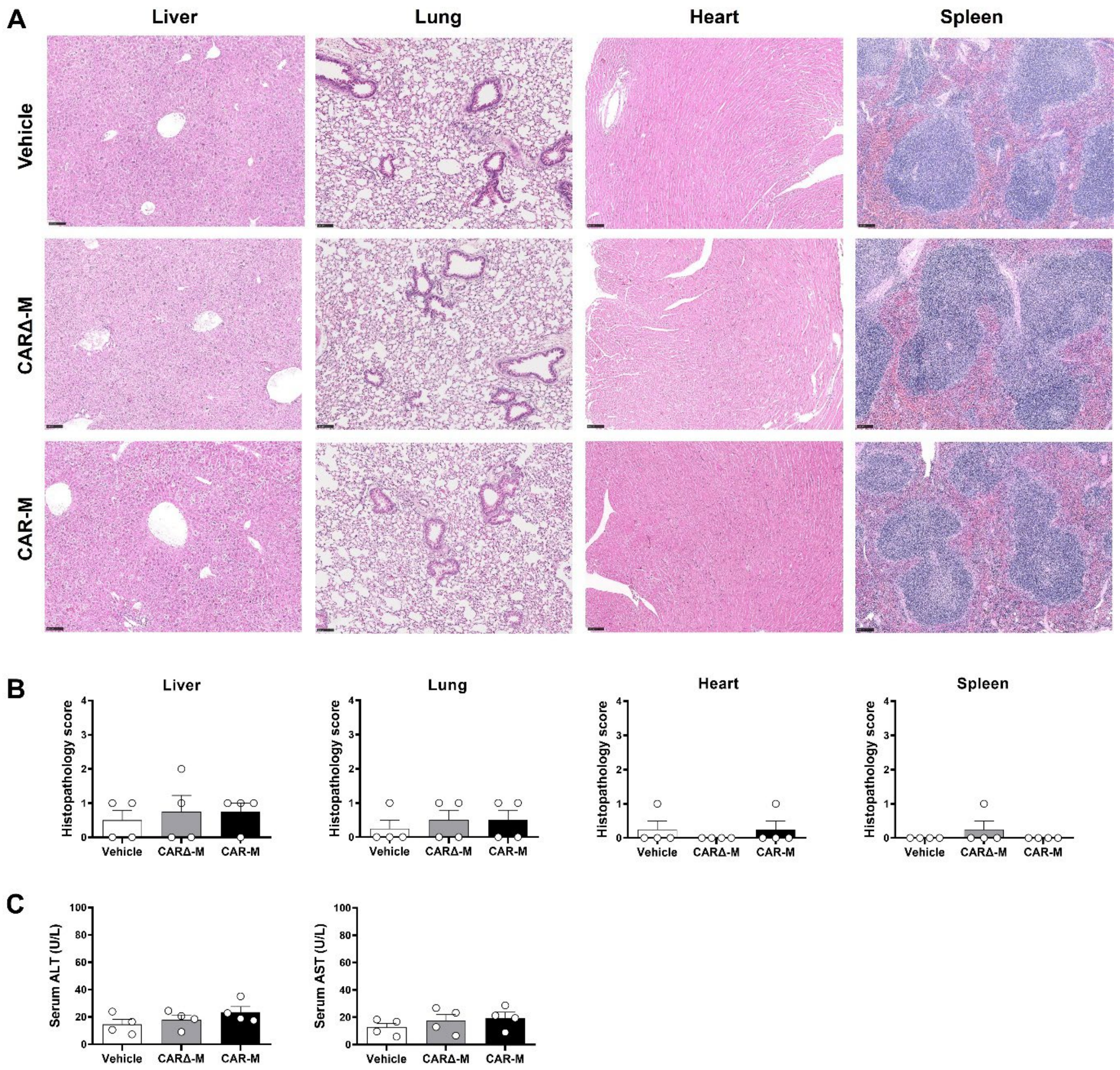

Supplementary Figure 3. CAR-M treatment does not affect other organs.

(A) H&E staining of livers, lungs, hearts and spleens from IRI mice treated with CARΔ-M or CAR-M. (B) The histopathology scores of the various tissues. (C) Serological markers of liver injury, alanine aminotransferase (ALT) and aspartate aminotransferase (AST). Data shown are the mean ± SEM (n=4 per group).

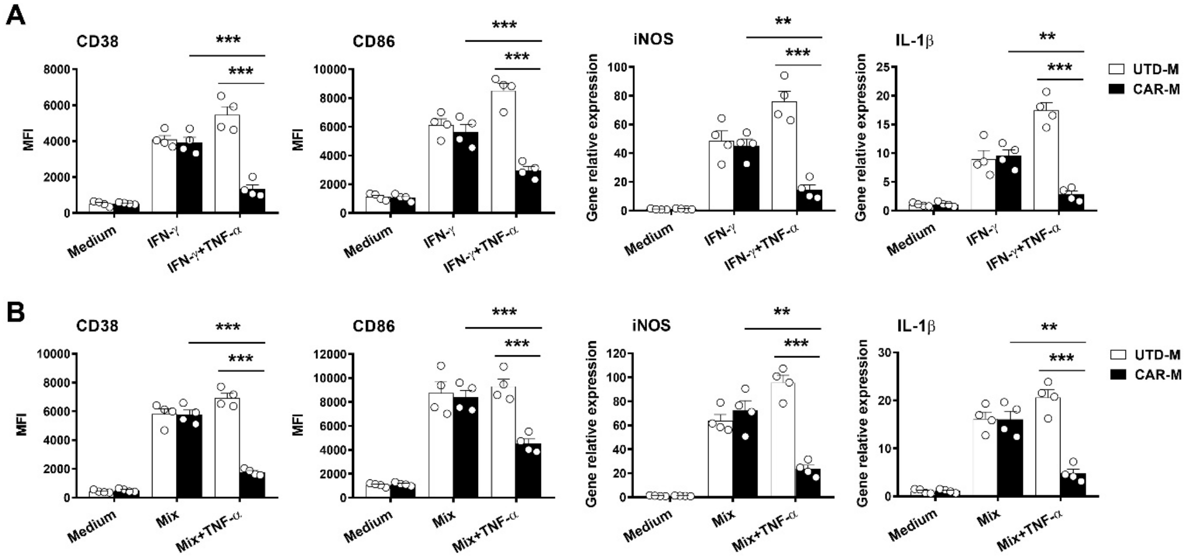

Supplementary Figure 4. CAR-Ms were resistant to M1-inducing cytokines in presence of TNF-α.

UTD-Ms and CAR-Ms incubated with medium or medium with IFN-γ (A) or mixture (B) of inflammatory cytokines (IFN-γ, IL-1β and IL-6) in the presence or absence of TNF-α for 24 hours. The M1-like phenotype of UTD-M or CAR-M was assessed by flow cytometry (CD38 and CD86) and quantitative PCR (iNOS and IL-1β). Data shown are the mean ± SEM (n=4 per group) and are representative of 3 independent experiments. **P<0.01, ***P<0.001.

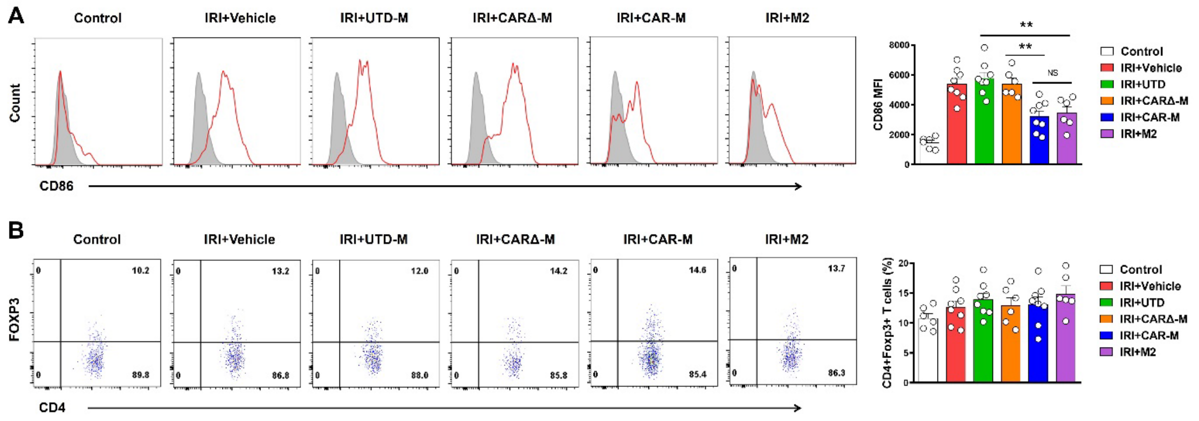

Supplementary Figure 5. CAR-Ms reduced M1 endogenous macrophages but did not induce Tregs in the kidney of IRI mice.

(A and B) Representative flow cytometric analysis of M1 markers CD86 on endogenous macrophages and Tregs (CD4+Foxp3+ cells) in kidneys from IRI mice treated with UTD-M, CAR**Δ**-M, CAR-M or M2. Data shown are the mean ± SEM (n=6-8 per group). NS: not significant, **P<0.01.

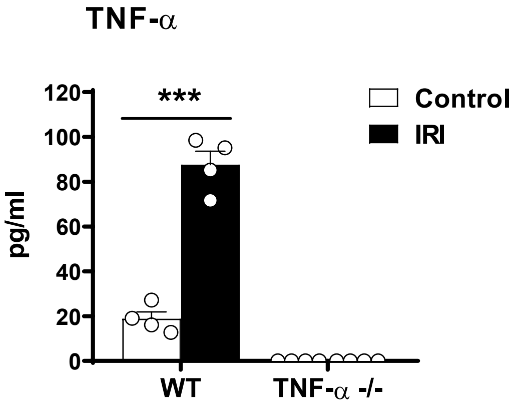

Supplementary Figure 6. The absence of TNF-α in TNF-α knockout mice with IRI.

The serum level of TNF-α was measured in WT or TNF-α knockout mice with or without IRI. Data shown are the mean ± SEM (n=4 per group). ***P<0.001.

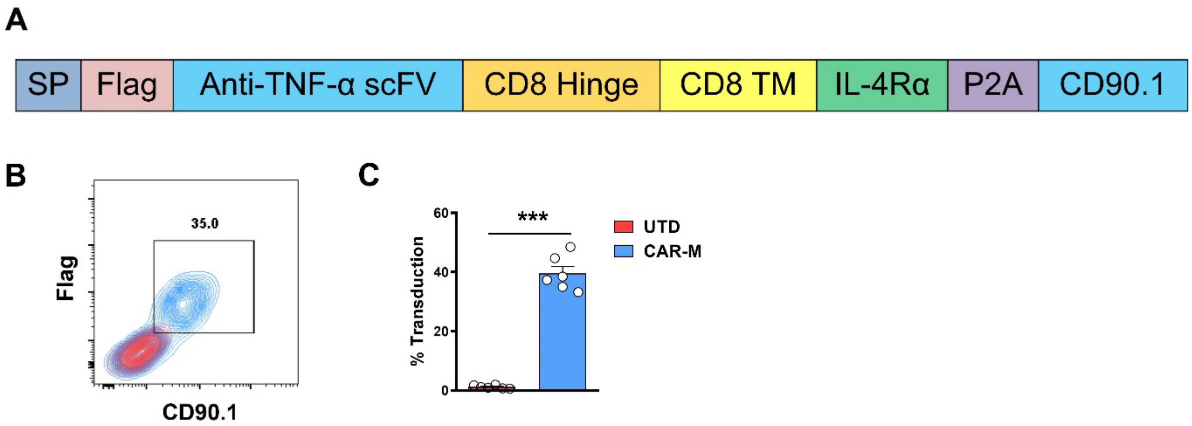

Supplementary Figure 7. CAR construct design for AN study.

(A) Schematic diagram of anti-TNF-α CAR construct design for AN study. The CAR contained a Flag tag epitope, a single-chain fragment variable (scFv) antibody specific for TNF-α, the hinge and transmembrane domains of mouse CD8α, and a mouse IL-4 receptor intracellular domain followed by a P2A flanked surface expression marker CD90.1, a transduction marker that also facilitates sorting of CAR-expressing cells from BALB/c. (B, C) Transduction efficiency was determined by staining for Flag-tag and CD90.1 expressed on the transduced macrophage. Representative flow plots (B) and a summary bar graph (C) are shown. Data shown are the mean ± SEM (n=6 per group). ***P<0.001.

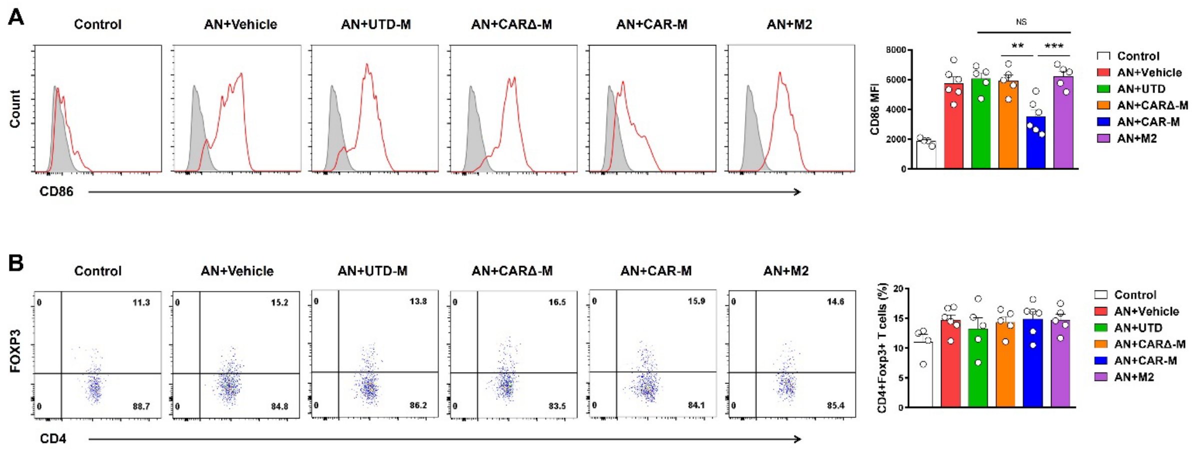

Supplementary Figure 8. S14. CAR-Ms reduced M1 endogenous macrophages but did not induce Tregs in the kidney of AN mice.

(A and B) Representative flow cytometric analysis of M1 marker CD86 on endogenous macrophages and Tregs (CD4+Foxp3+ cells) in kidneys from AN mice treated with UTD-M, CAR**Δ**-M, CAR-M or M2. Data shown are the mean ± SEM (n=4-6 per group). NS: not significant, **P<0.01.

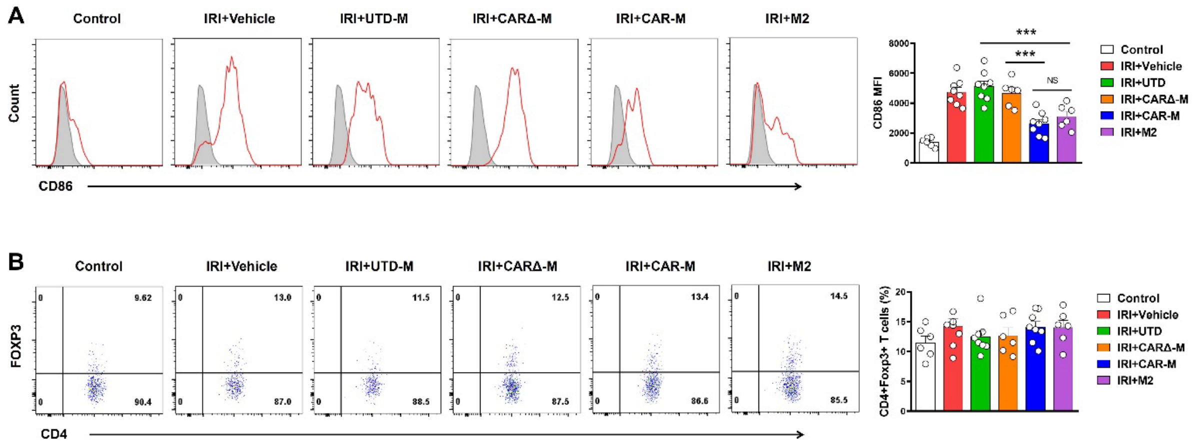

Supplementary Figure 9. CAR-Ms reduced M1 endogenous macrophages but did not induce Tregs in the liver of IRI mice.

(A and B) Representative flow cytometric analysis of M1 marker CD86 on endogenous macrophages and Tregs (CD4+Foxp3+ cells) in livers from IRI mice treated with UTD-M, CAR**Δ**-M, CAR-M or M2. Data shown are the mean ± SEM (n=4-6 per group). NS: not significant, ***P<0.001.

Supplementary Table 1. Real-time PCR primers

**Gene**	**Forward (5'-3')**	**Reverse (5'-3')**
Arginase (M)	agtctggcagttggaagcat	ctggttgtcaggggagtgtt
FIZZ1 (M)	tgctgggatgactgctactg	ctgggttctccacctcttca
IL-10 (M)	ccagtacagccgggaagaca	cagctggtcctttgtttgaaaga
Ym-1 (M)	cagctgggatcttcctacca	attctgcattccagcaaagg
iNOS (M)	caccttggagttcacccagt	accactcgtacttgggatgc
IFN-γ (M)	gcgtcattgaatcacacctg	acctgtgggttgttgacctc
TNF-α (M)	gctgagctcaaaccctggta	cggactccgcaaagtctaag
IL-1β (M)	tgccaccttttgacagtgatg	atgtgctgctgcgagatttg
IL-6 (M)	cacaagtccggagaggagac	ttgccattgcacaactcttt
CCL2 (M)	agcaccagccaactctcact	cgttaactgcatctggctga
CCL5 (M)	gacaccactccctgctgctt	acaaacacgactgcaagattgg
CXCL1 (M)	tggctgggattcacctcaagaaca	tgtggctatgacttcggtttgggt
CXCL2 (M)	acatcccacccacacagtgaaaga	acatcccacccacacagtgaaaga
TRAF1 (M)	gagcagacaacctccatcctgt	gaaggaacagccaacacctgca
TRAF2 (M)	acctgtgatggctgtggcaaga	tctgaacagccaacggtgtgga
TNFAIP3 (M)	agcaagtgcaggaaagctggct	gctttcgcagaggcagtaacag
TRAF1 (H)	cgatggcactttcctgtggaag	tacagccgcaggcacaacttgt
TRAF2 (H)	gagcagaaggtcttggagatgg	gcagacacatcttgtagccgtac
TNFAIP3 (H)	ctcaactggtgtcgagaagtcc	ttccttgagcgtgctgaacagc

M=mouse, H=human.

Nucleotide sequences of CAR constructs

Mouse TNF-α_CAR

GM-CSF – Flag – anti-TNFα scFV (ECD) – CD8a (TMD) – IL4Rα (ICD)

**ATGTGGCTGCAGAACCTGCTGTTTCTGGGCATTGTGGTGTACTCTCTCTCTGAC
TACAAGGACGATGATGACAAGGATTTGCAGCTGGTTGAGTCTGGAGGCGACTC
AGTGCAGCCTGGAAGGAGTCTGAAGCTGTCCTGCGTGGCCAGCGGCTTCACAT
TCTCAAACTACGACATGGCATGGGTGAGGCAGGCCCCCACCAAGGGACTGGAG
TGGGTGGCCTCAATCAGCCCAAACGGCGACAGGACCTATTATAGACAGTCAGT
GAAGGGGAGATTTACAGTGTCCAGGGACAACAGCAAGTCTACCCTCTACCTGC
AGATGGACAGCCTGAGGAGCGAGGACACCGCCACATATTACTGCACCAGACAG
GGCGCCGCCCTGATCCTGATGGATGCATGGGGGCAGGGCGCCAGTGTGACAG
TGTCCTCCGGTGGAGGAGGATCAGGGGGCGGGGGAGCGGCGGAGGGGGCA
GTGATATTGTGCTGACCCAGTCCCCTGTGCTGGCCGTGTCCCTGGGCCAGAGA
GCCACCATCAGCTGTAGAGCTAGTCAGAGCGTGTCCTTCAGCAGCATTTCCCT
GATGCATTGGTACCAGCAGAAGCCAGGCCAGCAGCCCAAGCTGCTGATTTATC
GCGCATCCAACCTGGCTTCCGGCATCCCTGCCAGATTTAGCGGCTCCGGATCC
GGCACAGACTTCACACTGACTATCGACCCCGTGCAAGCCGACGACATCGCCGC
ATACTTCTGCCAGCAGAGTAGAGAGTTCCCTCTGACCTTTGGAAGTGGCACCA
AGCTGGAATTCAAGACAACCACACCTGCCCCACGGCCACCCACCCCAGCCCCC
ACAATCGCCTCTCAGCCACTGTCCCTGAGACCTGAAGCTTGTCGGCCAGCCGC
CGGCGGGGCCGTGCACACTAGAGGCCTGGACTTTGCTTGCGACATCTACATCT
GGGCTCCACTCGCCGGCACATGCGGCGTGCTTCTGCTGTCTCTGGTGATCACA
CTGTACTGCAAGATCAAGAAGATCTGGTGGGACCAGATCCCTACACCTGCACG
GTCCCCTCTGGTCGCTATTATAATCCAGGACGCCCAGGTGCCCCTGTGGGACA
AGCAAACAAGAAGTCAGGAGTCCACCAAGTATCCACACTGGAAGACTTGCCTG
GATAAACTACTGCCATGTCTGCTGAAGCACAGAGTGAAGAAAAAGACTGATTT
CCCTAAGGCCGCCCCAACAAAGAGCCTGCAGTCACCAGGCAAAGCCGGCTGGT
GTCCTATGGAGGTGTCCAGGACAGTTCTGTGGCCAGAGAACGTGAGCGTCAGC
GTGGTGCGATGCATGGAGCTGTTTGAGGCCCCAGTCCAGAACGTGGAAGAGGA
GGAAGACGAGATTGTCAAGGAAGATCTGAGCATGTCACCAGAGAATAGCGGAG**

GTTGCGGTTTTCAGGAGAGCCAGGCTGATATCATGGCAAGGTTGACTGAAAAT
CTGTTCAGCGACCTGCTGGAAGCCGAGAATGGAGGACTGGGGCAGTCCGCCCT
GGCCGAGTCTTGTTCTCCTCTGCCATCTGGATCCGGGCAAGCTTCAGTGTCTTG
GGCTTGTCTGCCTATGGGCCCTTCAGAGGAGGCCACCTGCCAGGTGACAGAGC
AGCCTTCCCACCCTGGCCCACTGAGCGGGTCCCCAGCCCAGAGCGCCCCAACC
CTGGCTTGTACCCAGGTGCCTCTCGTGCTGGCTGACAATCCCGCCTATCGCTCT
TTCTCTGACTGTTGTTCACCTGCTCCAAATCCAGGCGAGCTGGCCCCTGAACAG
CAGCAGGCCGACCACCTGGAGGAGGAGGAGCCCCCTAGCCCAGCCGACCCAC
ACTCTAGCGGCCCTCCAATGCAGCCAGTGGAGAGCTGGGAACAGATCCTGCAC
ATGTCCGTACTGCAGCACGGCGCGGCCGCTGGTAGCACACCTGCACCAGCTGG
CGGATACCAGGAGTTCGTGCAGGCCGTGAAACAGGGTGCTGCCCAGGATCCCG
GAGTGCCCGGGGTGAGACCCAGCGGTGACCCAGGATATAAGGCCTTTAGCTCC
CTGCTGAGCTCTAACGGAATCCGGGGAGACACCGCTGCCGCCGGCACCGACGA
CGGGCACGGAGGGTATAAGCCATTCCAGAATCCAGTCCCTAATCAGAGCCCCT
CAAGCGTGCCTCTGTTCACTTTTGGACTGGACACCGAGCTGTCACCTTCTCCCC
TGAACTCCGACCCGCCTAAGTCTCCACCAGAGTGCCTGGGGCTGGAGCTGGGC
CTGAAAGGCGGAGACTGGGTGAAGGCCCCCCCACCAGCCGACCAGGTGCCCA
AGCCTTTCGGAGATGATCTGGGCTTCGGCATCGTCTATAGCTCACTGACCTGCC
ACCTGTGTGGCCATCTGAAACAGCACCACAGCCAGGAAGAAGGCGGCCAGAGC
CCCATCGTGGCCTCACCCGGTTGTGGTTGCTGTTACGACGATAGATCACCATCA
CTGGGCTCTCTGAGCGGAGCCCTGGAGAGCTGTCCTGAGGGGATCCCCCCTGA
AGCTAACCTGATGTCTGCCCCTAAGACACCATCTAACCTGTCCGGAGAAGGCA
AAGGCCCAGGCCACAGCCCTGTTCCCTCTCAGACAACTGAGGTGCCCGTGGGC
GCTCTCGGAATCGCAGTGTCTTGA

Mouse TNF-α_CARΔ

GM-CSF – Flag – anti-TNFα scFV (ECD) – CD8a (TMD)

ATGTGGCTGCAGAACCTGCTGTTCCTGGGCATCGTGGTGTACTCCCTGTCCGA
TTACAAGGACGACGACGATAAGGACCTGCAGCTGGTGGAGAGTGGAGGGGAC
TCTGTGCAGCCTGGCAGGTCTCTGAAGCTGAGCTGTGTCGCCTCTGGATTTAC
ATTCTCCAATTATGACATGGCCTGGGTGCGGCAGGCACCCACAAAGGGTCTGG

**AATGGGTGGCAAGCATTTCTCCTAACGGCGACCGGACCTATTACAGGCAGTCC
GTGAAGGGCCGCTTCACAGTGAGCCGAGATAATTCTAAAAGCACACTGTATCT
GCAGATGGATAGCCTCAGATCTGAGGACACAGCCACTTACTATTGTACCAGAC
AGGGCGCAGCTCTGATACTGATGGACGCCTGGGGACAGGGCGCATCCGTGACC
GTGAGTTCCGGCGGGGGCGGGAGCGGCGGCGGCGGAAGCGGGGGCGGGGGA
TCCGACATCGTGCTGACTCAGAGTCCCGTGCTGGCTGTGTCCCTGGGCCAGCG
CGCAACTATTTCCTGCAGGGCCTCACAGTCTGTGAGCTTTAGCTCCATTTCACT
GATGCACTGGTACCAGCAGAAGCCCGGACAGCAGCCCAAACTGCTGATCTACA
GAGCTAGTAACCTGGCTTCAGGCATCCCCGCCAGATTCAGCGGCAGCGGCAGC
GGGACTGACTTCACACTGACTATCGACCCCGTGCAGGCTGACGACATCGCCGC
CTACTTCTGTCAGCAGTCAAGAGAATTTCCCCTGACCTTCGGCAGCGGCACAAA
GCTGGAGTTTAAGACTACAACCCCAGCCCCTAGGCCCCCAACTCCTGCCCCAA
CCATCGCATCTCAGCCACTGTCCCTGAGGCCTGAGGCCTGTAGACCCGCCGCT
GGAGGGGCCGTGCACACCAGAGGCCTGGACTTCGCCTGTGACATCTACATTTG
GGCCCCCCTGGCCGGCACTTGCGGCGTGCTGCTGCTGTCCCTGGTGATCACCC
TGTACTGCTGA**

Mouse TNF-α_CAR (CD90.1)

GM-CSF – Flag – anti-TNFα scFV (ECD) – CD8a (TMD) – IL4Rα (ICD) – CD90.1

**ATGTGGCTGCAGAACCTCCTGTTCCTGGGAATTGTGGTGTACAGCCTGAGCGA
TTACAAAGATGACGACGACAAGGATCTGCAGCTTGTGGAAAGTGGAGGAGACA
GTGTGCAGCCTGGCAGAAGCCTGAAGCTCTCATGCGTGGCCTCAGGCTTCACA
TTCAGTAATTACGACATGGCCTGGGTGCGGCAGGCCCCTACTAAGGGCCTGGA
GTGGGTGGCCAGCATTTCTCCTAACGGCGATAGGACCTACTACAGGCAGAGCG
TGAAGGGCCGCTTCACCGTGTCCCGCGATAATTCCAAATCCACACTGTACCTCC
AGATGGATAGTCTGAGAAGCGAGGACACAGCCACTTACTATTGTACAAGACAG
GGTGCCGCCCTGATCCTGATGGACGCATGGGGCCAAGGCGCTAGCGTGACCGT
GAGCAGCGGGGGCGGCGGATCCGGGGGCGGAGGGAGCGGTGGCGGCGGATC
TGACATCGTGCTGACCCAGTCTCCTGTGCTGGCAGTGTCTCTGGGACAGAGAG
CTACTATTTCTTGTCGCGCATCTCAGTCCGTGAGCTTCTCCTCTATCTCCCTGA
TGCACTGGTACCAGCAGAAGCCCGGCCAGCAGCCTAAGCTGCTGATTTACAGA**

GCCAGCAATCTGGCTAGCGGCATCCCTGCCAGATTCTCTGGAAGTGGATCCGG

CACCGACTTCACCCTGACTATCGACCCTGTGCAGGCCGACGACATCGCTGCAT

ACTTCTGCCAGCAGAGCAGGGAATTCCCTCTGACTTTCGGCTCCGGCACCAAG

CTGGAATTCAAGACCACAACCCCTGCTCCAAGACCACCCACCCCCGCCCCAAC

AATCGCTAGCCAGCCACTGAGCCTGAGGCCAGAGGCCTGCAGACCAGCTGCCG

GCGGCGCCGTGCACACTAGAGGCCTGGACTTTGCTTGCGACATTTACATTTGG

GCCCCCCTGGCTGGAACCTGCGGGGTGCTGCTGCTGAGCCTCGTGATAACCCT

GTATTGTAAGATTAAGAAAATTTGGTGGGACCAGATCCCTACCCCTGCCAGATC

ACCCCTGGTGGCCATTATCATCCAGGACGCACAGGTGCCACTGTGGGACAAAC

AGACCCGCTCTCAGGAGAGCACCAAGTACCCCCACTGGAAGACATGCCTGGAT

AAGCTGCTGCCCTGCCTGCTGAAACACAGAGTGAAAAAGAAGACAGACTTCCC

CAAAGCCGCTCCTACAAAGTCCCTGCAGTCCCCCGGAAAGGCCGGGTGGTGCC

CCATGGAAGTGTCCAGAACAGTGCTGTGGCCTGAAAACGTGTCTGTGTCCGTG

GTCAGATGCATGGAGCTGTTTGAAGCCCCCGTGCAGAACGTGGAGGAGGAGGA

GGACGAAATCGTGAAAGAGGATCTGAGCATGTCTCCAGAAAATAGCGGCGGCT

GCGGATTCCAGGAGAGCCAGGCCGATATTATGGCCAGACTTACCGAGAACCTG

TTCTCCGATCTGCTCGAGGCCGAGAACGGAGGGCTGGGGCAGTCCGCCCTGGC

CGAGTCTTGTTCCCCACTGCCCTCCGGCAGCGGACAGGCTTCTGTGAGCTGGG

CCTGCCTGCCCATGGGCCCTTCTGAGGAGGCCACTTGCCAGGTGACAGAGCAG

CCATCACACCCCGGCCCCCTGAGCGGCAGTCCTGCTCAGAGCGCCCCTACCCT

CGCCTGCACCCAGGTGCCTCTCGTGCTCGCTGATAACCCTGCCTACAGATCCTT

CAGCGATTGCTGTTCCCCAGCTCCCAACCCAGGCGAGCTGGCTCCCGAGCAGC

AGCAGGCCGACCACCTGGAGGAGGAAGAGCCTCCAAGTCCAGCTGATCCTCAC

AGCTCCGGGCCTCCCATGCAGCCTGTGGAGTCCTGGGAGCAGATCCTGCACAT

GAGCGTGCTGCAGCACGGCGCCGCCGCTGGGTCTACCCCTGCCCCCGCAGGC

GGATATCAGGAGTTTGTGCAGGCCGTGAAGCAGGGCGCTGCTCAAGACCCTGG

GGTGCCTGGGGTGAGGCCCTCCGGAGATCCTGGATATAAGGCCTTCAGTAGCC

TTCTGTCTTCCAACGGGATCAGAGGCGACACTGCCGCAGCCGGAACAGACGAC

GGCCACGGCGGCTACAAGCCTTTCCAGAACCCTGTGCCTAACCAGAGCCCCTC

TAGCGTGCCACTGTTCACCTTCGGACTGGACACCGAGCTGAGCCCTTCACCAC

TAAACTCTGATCCACCCAAGAGCCCTCCCGAATGTCTGGGACTGGAACTGGGA

CTGAAGGGCGGCGACTGGGTGAAGGCTCCTCCTCCCGCTGACCAGGTGCCTAA
GCCCTTCGGGGACGACCTGGGATTCGGAATCGTGTACTCAAGCCTGACATGCC
ATCTGTGTGGGCACCTGAAGCAGCATCACTCCCAGGAGGAGGGCGGACAGAGC
CCCATTGTGGCCAGCCCTGGCTGTGGATGCTGTTATGATGACAGAAGCCCAAG
TCTGGGAAGCCTGTCTGGCGCACTGGAAAGCTGCCCTGAGGGGATTCCCCCTG
AGGCCAATCTGATGTCTGCCCCCAAGACTCCCTCCAACCTGTCAGGCGAGGGT
AAGGGCCCTGGCCACTCTCCCGTGCCCAGTCAGACAACTGAGGTTCCCGTGGG
GGCCCTCGGCATTGCTGTGTCAGCCACTAACTTTAGCCTGCTAAAACAGGCAG
GTGACGTGGAGGAGAATCCTGGCCCAATGAATCCAGCTATCAGCGTGGCTCTG
CTGCTGAGCGTGCTGCAGGTCTCTCGGGGCCAGAAGGTGACCAGCCTGACCGC
CTGCCTGGTGAACCAGAATCTGCGACTCGACTGTCGGCATGAGAACAATACTA
AGGATAACAGCATCCAGCACGAATTTAGTCTGACTAGGGAGAAGAGAAAGCAC
GTGCTGTCTGGAACACTTGGCATCCCTGAGCACACCTATCGGTCCAGAGTGAC
CCTGAGCAACCAGCCCTACATCAAGGTCCTGACTCTGGCCAACTTTACAACAAA
GGACGAGGGCGATTACTTCTGCGAGCTGAGGGTGTCCGGAGCTAATCCCATGT
CATCTAATAAGTCCATCAGCGTGTACAGGGATAAGCTGGTGAAGTGCGGAGGC
ATCAGCCTGCTGGTCCAGAACACATCTTGGATGCTCCTGCTCCTGCTGTCCCTG
TCTCTGCTGCAGGCTCTGGATTTCATTAGTCTGTGA

Mouse TNF-α_CARΔ (CD90.1)

GM-CSF – Flag – anti-TNFα scFV (ECD) – CD8a (TMD) – CD90.1

ATGTGGCTGCAGAACCTGCTGTTCCTGGGCATCGTGGTGTACTCCCTGTCCGA
TTACAAGGACGACGACGATAAGGACCTGCAGCTGGTGGAGAGTGGAGGGGAC
TCTGTGCAGCCTGGCAGGTCTCTGAAGCTGAGCTGTGTCGCCTCTGGATTTAC
ATTCTCCAATTATGACATGGCCTGGGTGCGGCAGGCACCCACAAAGGGTCTGG
AATGGGTGGCAAGCATTTCTCCTAACGGCGACCGGACCTATTACAGGCAGTCC
GTGAAGGGCCGCTTCACAGTGAGCCGAGATAATTCTAAAAGCACACTGTATCT
GCAGATGGATAGCCTCAGATCTGAGGACACAGCCACTTACTATTGTACCAGAC
AGGGCGCAGCTCTGATACTGATGGACGCCTGGGGACAGGGCGCATCCGTGACC
GTGAGTTCCGGCGGGGGCGGGAGCGGCGGCGGCGGAAGCGGGGGCGGGGGA
TCCGACATCGTGCTGACTCAGAGTCCCGTGCTGGCTGTGTCCCTGGGCCAGCG

CGCAACTATTTCCTGCAGGGCCTCACAGTCTGTGAGCTTTAGCTCCATTTCACT

GATGCACTGGTACCAGCAGAAGCCCGGACAGCAGCCCAAACTGCTGATCTACA

GAGCTAGTAACCTGGCTTCAGGCATCCCCGCCAGATTCAGCGGCAGCGGCAGC

GGGACTGACTTCACACTGACTATCGACCCCGTGCAGGCTGACGACATCGCCGC

CTACTTCTGTCAGCAGTCAAGAGAATTTCCCCTGACCTTCGGCAGCGGCACAAA

GCTGGAGTTTAAGACTACAACCCCAGCCCCTAGGCCCCCAACTCCTGCCCCAA

CCATCGCATCTCAGCCACTGTCCCTGAGGCCTGAGGCCTGTAGACCCGCCGCT

GGAGGGGCCGTGCACACCAGAGGCCTGGACTTCGCCTGTGACATCTACATTTG

GGCCCCCCTGGCCGGCACTTGCGGCGTGCTGCTGCTGTCCCTGGTGATCACCC

TGTACTGCGCCACTAACTTTAGCCTGCTAAAACAGGCAGGTGACGTGGAGGAG

AATCCTGGCCCAATGAATCCAGCTATCAGCGTGGCTCTGCTGCTGAGCGTGCT

GCAGGTCTCTCGGGGCCAGAAGGTGACCAGCCTGACCGCCTGCCTGGTGAACC

AGAATCTGCGACTCGACTGTCGGCATGAGAACAATACTAAGGATAACAGCATC

CAGCACGAATTTAGTCTGACTAGGGAGAAGAGAAAGCACGTGCTGTCTGGAAC

ACTTGGCATCCCTGAGCACACCTATCGGTCCAGAGTGACCCTGAGCAACCAGC

CCTACATCAAGGTCCTGACTCTGGCCAACTTTACAACAAAGGACGAGGGCGAT

TACTTCTGCGAGCTGAGGGTGTCCGGAGCTAATCCCATGTCATCTAATAAGTCC

ATCAGCGTGTACAGGGATAAGCTGGTGAAGTGCGGAGGCATCAGCCTGCTGGT

CCAGAACACATCTTGGATGCTCCTGCTCCTGCTGTCCCTGTCTCTGCTGCAGGC

TCTGGATTTCATTAGTCTGTGA

Human TNF-α_CAR

GM-CSF – Flag – anti-TNFα scFV (ECD) – CD8a (TMD) – IL4Rα (ICD)

ATGTGGCTGCAGTCACTCCTGCTGCTGGGCACAGTGGCCTGTTCTATTAGCGA

CTACAAAGATGACGACGATAAGGAGGTCAAGCTCGAGGAGTCCGGCGGAGGC

CTGGTCCAGCCTGGAGGATCAATGAAACTGTCCTGCGTCGCCAGCGGCTTCAT

CTTTAGCAATCACTGGATGAATTGGGTGCGGCAGTCCCCCGAGAAGGGACTGG

AGTGGGTGGCCGAGATCCGATCTAAGAGCATCAACTCCGCCACCCACTATGCC

GAGAGCGTGAAGGGTCGCTTCACAATCAGTAGAGATGATAGCAAATCCGCTGT

GTACCTGCAAATGACTGATTTGAGAACTGAGGACACAGGCGTGTATTACTGCA

GCCGCAATTATTATGGAAGCACATATGATTACTGGGGCCAGGGCACCACACTG

ACCGTGTCCTCCGGAGGTGGCGGTAGTGGCGGCGGGGGTTCTGGGGGCGGGG
GCAGCGATATTCTGCTGACACAGTCTCCTGCCATCCTGTCTGTGTCTCCTGGCG
AGCGCGTGTCCTTCAGCTGTCGCGCCTCTCAGTTTGTGGGATCTAGCATACACT
GGTACCAGCAGCGGACAAATGGCAGCCCAAGGCTGCTGATTAAATACGCTAGC
GAGTCTATGAGCGGTATTCCAAGTAGGTTCTCAGGCTCAGGGTCCGGCACCGA
CTTTACACTGAGCATTAACACAGTGGAGTCCGAGGACATCGCCGATTATTATTG
CCAGCAGTCCCACTCCTGGCCTTTTACATTTGGCTCTGGCACCAACCTGGAAGT
GAAGACAACTACCCCCGCACCTAGGCCTCCCACACCAGCCCCCACCATCGCAT
CCCAGCCTTTGTCCCTCAGACCGGAGGCCTGTAGGCCAGCCGCCGGGGGCGCC
GTGCATACAAGAGGCCTGGACTTTGCCTGCGACATTTACATCTGGGCTCCCCT
GGCTGGGACCTGTGGCGTGCTGCTGCTGAGCCTGGTGATCACCCTGTACTGTA
AGATCAAGAAGGAGTGGTGGGATCAAATCCCTAACCCTGCTCGGTCTAGGCTG
GTGGCCATTATTATCCAGGATGCTCAGGGCTCCCAGTGGGAAAAGCGGAGCAG
GGGGCAGGAACCAGCCAAATGTCCACACTGGAAGAACTGTCTGACCAAGCTGC
TGCCCTGCTTCCTGGAGCACAATATGAAGCGGGACGAAGACCCACACAAAGCC
GCTAAGGAAATGCCCTTTCAGGGCAGCGGCAAAAGCGCCTGGTGCCCTGTGGA
AATCTCAAAGACAGTGCTGTGGCCCGAGAGCATCAGCGTGGTGCGGTGCGTGG
AACTGTTTGAGGCACCCGTGGAGTGTGAGGAAGAGGAGGAGGTCGAGGAGGA
GAAAGGAAGCTTTTGTGCCAGCCCAGAAAGTTCCAGAGACGATTTTCAGGAGG
GCCGGGAGGGCATTGTGGCAAGACTGACCGAGTCTCTGTTCCTGGACCTGCTG
GGAGAGGAAAACGGGGGCTTCTGCCAGCAGGACATGGGCGAGAGCTGCCTGC
TCCCACCTTCAGGATCCACTAGCGCCCACATGCCCTGGGACGAATTTCCATCTG
CCGGCCCAAAGGAGGCCCCCCCATGGGGAAAAGAGCAGCCACTCCATCTGGAG
CCCAGCCCCCCTGCCAGTCCAACCCAGAGCCCCGACAATCTGACCTGTACAGA
GACCCCACTGGTCATTGCCGGTAACCCAGCTTACAGGAGCTTTAGCAATAGCC
TGAGCCAGTCCCCTTGTCCCAGAGAGCTGGGCCCTGACCCACTGCTGGCCCGG
CATCTGGAGGAGGTGGAGCCAGAAATGCCCTGCGTGCCACAACTGAGCGAGCC
CACAACCGTGCCTCAGCCCGAACCAGAGACCTGGGAGCAGATTTTGAGACGGA
ACGTGCTGCAGCACGGGGCCGCCGCCGCCCCTGTGTCTGCCCCCACCTCTGGG
TATCAGGAATTTGTGCATGCCGTGGAGCAGGGAGGCACACAGGCTAGCGCTGT
CGTGGGCCTGGGACCCCCCGGCGAAGCCGGGTACAAGGCCTTTAGCAGCCTGC

TGGCCTCCTCTGCCGTGAGCCCCGAGAAGTGTGGCTTCGGTGCAAGCAGCGGC
GAGGAGGGATACAAGCCATTCCAGGATCTGATCCCTGGCTGTCCAGGCGATCC
CGCCCCTGTGCCCGTGCCTCTGTTCACCTTCGGCCTGGACCGGGAGCCACCTA
GGAGTCCCCAGAGCAGCCATTTGCCCAGCAGCAGCCCAGAACATCTGGGACTG
GAGCCTGGAGAGAAGGTGGAGGATATGCCTAAGCCTCCACTCCCTCAGGAGCA
GGCCACCGATCCTCTGGTGGACTCCCTGGGCTCAGGCATCGTGTACTCTGCCC
TGACATGCCATCTGTGTGGCCATCTGAAGCAGTGCCACGGCCAGGAGGATGGA
GGACAGACCCCCGTGATGGCATCTCCATGTTGCGGGTGCTGCTGCGGGGACCG
ATCTTCACCTCCCACAACCCCTCTGAGAGCCCCCGATCCCTCCCCTGGCGGAGT
GCCTCTGGAAGCCTCTCTGTGTCCTGCCAGTCTGGCCCCCTCAGGCATCTCCG
AGAAGAGTAAATCAAGTTCAAGCTTCCACCCCGCCCCAGGGAACGCCCAGTCC
TCCTCCCAGACCCCAAAGATTGTGAATTTTGTGAGTGTGGGACCCACCTATATG
AGAGTGTCATGA

Human TNF-α_CARΔ

GM-CSF – Flag – anti-TNFα scFV (ECD) – CD8a (TMD)

ATGTGGCTGCAGTCTCTGCTGCTGCTGGGCACTGTGGCCTGTAGCATTAGCGA
TTATAAAGATGATGATGACAAGGAAGTGAAGCTGGAGGAGAGCGGGGGCGGA
CTGGTGCAGCCTGGAGGGTCCATGAAGCTGAGCTGCGTGGCCTCTGGGTTTAT
CTTCTCAAACCACTGGATGAACTGGGTGAGGCAGAGCCCCGAGAAGGGCCTGG
AGTGGGTGGCCGAGATCAGGTCCAAGTCAATTAATAGCGCAACACACTACGCC
GAGAGCGTGAAGGGGAGATTCACCATCAGTAGGGACGACTCCAAGAGCGCTGT
GTACCTGCAGATGACCGACCTGCGAACCGAAGATACTGGCGTGTACTACTGTT
CTAGGAACTATTATGGGAGCACATACGATTATTGGGGACAGGGCACCACTCTG
ACTGTCTCCTCAGGCGGCGGAGGCAGCGGCGGAGGGGGAAGCGGCGGGGGAG
GGAGCGACATCCTGCTGACCCAGAGCCCCGCAATCCTGAGCGTGTCCCCCGGC
GAGAGAGTGAGCTTCAGCTGCAGGGCCAGCCAGTTTGTGGGATCCAGCATCCA
CTGGTACCAGCAGCGGACTAATGGATCCCCACGGCTGCTCATCAAGTATGCCT
CCGAGAGCATGTCAGGCATCCCAAGCAGGTTCAGCGGAAGCGGCAGCGGCAC
CGACTTTACACTGAGCATTAATACAGTGGAGTCTGAGGACATCGCTGACTATTA
TTGTCAGCAGAGCCACAGCTGGCCCTTCACCTTCGGGTCTGGAACCAATCTGG

**AGGTGAAAACTACTACCCCCGCACCCCGGCCTCCAACCCCAGCCCCAACAATT
GCCAGCCAGCCGCTGTCCCTGAGACCTGAGGCCTGCAGACCAGCCGCCGGCG
GCGCCGTGCACACCAGAGGCCTGGATTTCGCCTGTGATATCTACATTTGGGCC
CCCCTGGCCGGGACCTGCGGAGTGCTGCTCCTGAGCCTGGTGATCACACTGTA
CTGTTGA**
