# Supplementary material for: Targeting inflammation with chimeric antigen receptor macrophages using a signal switch
Source: Nat Biomed Eng. 2025 May 7;9(9):1502–16. doi: 10.1038/s41551-025-01387-8 (PMC12443588; doi:10.1038/s41551-025-01387-8)
Supplement: Supplementary file 2 — Reporting Summary [file 41551_2025_1387_MOESM2_ESM.pdf]

nature portfolio

Corresponding author(s): Qi Cao

Last updated by author(s): Sep 22, 2024

Reporting Summary

Nature Portfolio wishes to improve the reproducibility of the work that we publish. This form provides structure for consistency and transparency in reporting. For further information on Nature Portfolio policies, see our Editorial Policies and the Editorial Policy Checklist.

Statistics

For all statistical analyses, confirm that the following items are present in the figure legend, table legend, main text, or Methods section.

n/a	Confirmed	
☐	☒	The exact sample size (*n*) for each experimental group/condition, given as a discrete number and unit of measurement
☐	☒	A statement on whether measurements were taken from distinct samples or whether the same sample was measured repeatedly
☐	☒	The statistical test(s) used AND whether they are one- or two-sided<br>*Only common tests should be described solely by name; describe more complex techniques in the Methods section.*
☒	☐	A description of all covariates tested
☐	☒	A description of any assumptions or corrections, such as tests of normality and adjustment for multiple comparisons
☐	☒	A full description of the statistical parameters including central tendency (e.g. means) or other basic estimates (e.g. regression coefficient) AND variation (e.g. standard deviation) or associated estimates of uncertainty (e.g. confidence intervals)
☐	☒	For null hypothesis testing, the test statistic (e.g. *F*, *t*, *r*) with confidence intervals, effect sizes, degrees of freedom and *P* value noted<br>*Give P values as exact values whenever suitable.*
☒	☐	For Bayesian analysis, information on the choice of priors and Markov chain Monte Carlo settings
☒	☐	For hierarchical and complex designs, identification of the appropriate level for tests and full reporting of outcomes
☒	☐	Estimates of effect sizes (e.g. Cohen's *d*, Pearson's *r*), indicating how they were calculated

Our web collection on statistics for biologists contains articles on many of the points above.

Software and code

Policy information about availability of computer code

Data collection	BD LSRFortessa flow cytometer was used to run samples and data was acquired and analyzed by Flowjo 10.8.0 software. In vivo biodistribution of XenoLight DiR-labeled macrophages was assessed and analyzed with the IVIS Spectrum In Vivo Imaging System (PerkinElmer). The RNA-seq sequencing libraries were sequenced on the Novaseq X platform (Illumina).
Data analysis	RNA sequencing data analysis: Library sequencing quality was determined using FastQC (Babraham Bioinformatics: www.bioinformatics.babraham.ac.uk/). Illumina adaptor sequence and low quality read trimming (read pair removed if < 20 base pairs) was performed using Trim Galore (Babraham Bioinformatics). STAR was used to align reads to mouse genome mm10 using ENSEMBL gene annotations as a guide. Read counts data corresponding to ENSEMBL gene annotations were generated the STAR flag --quantMode GeneCounts. Multiqc was used to verify quality metrics. All analyses were performed in the R Statistical Environment with tidyverse. Briefly, counts data were background corrected and normalized for library size using edgeR. DGE was determined using the QLFtest (BH MTC p <0.05). Gene lists were functionally annotated with Gene Ontology (GO), REACTOME and Kyoto Encyclopedia of Genes and Genomes (KEGG) pathways (adjusted p value <0.05) using the cluster Profiler package. Flow cytometry data were analyzed with Flowjo software. GraphPad Prism were used for data analysis.

For manuscripts utilizing custom algorithms or software that are central to the research but not yet described in published literature, software must be made available to editors and reviewers. We strongly encourage code deposition in a community repository (e.g. GitHub). See the Nature Portfolio guidelines for submitting code & software for further information.

Data

Policy information about availability of data

All manuscripts must include a data availability statement. This statement should provide the following information, where applicable:

- Accession codes, unique identifiers, or web links for publicly available datasets
- A description of any restrictions on data availability
- For clinical datasets or third party data, please ensure that the statement adheres to our policy

The main data supporting the results in the study are available within the paper and its Supplementary Information. RNA sequencing data that support the findings of this study will be deposited and made publicly available in the NCBI Gene Expression Omnibus repository.

Research involving human participants, their data, or biological material

Policy information about studies with human participants or human data. See also policy information about sex, gender (identity/presentation), and sexual orientation and race, ethnicity and racism.

Reporting on sex and gender	One of three donors is of female sex in PBMC dataset. Gender identity was not reported.
Reporting on race, ethnicity, or other socially relevant groupings	N/A
Population characteristics	Adult male and female participants, age 18–65 without diagnosis of blood disorders, such as leukemia and haemophilia, will be recruited through outpatient clinics at Westmead hospital. No personal information will be collected from participants. Blood sample will be collected when patients are having blood taken as part of their routine clinical care.
Recruitment	The potential participants will initially be approached through outpatient clinics at Westmead hospital by a member of the direct clinical care team. When the potential participant is approached, he/she will be provided with written participant information and consent forms and the study investigator will discuss the study and the contents of the written information and consent forms verbally with the potential participant.
Ethics oversight	All experiments with human samples were performed in accordance with protocols approved by Human Research Ethics Committee of Western Sydney Local Health District (ETH00071).

Note that full information on the approval of the study protocol must also be provided in the manuscript.

Field-specific reporting

Please select the one below that is the best fit for your research. If you are not sure, read the appropriate sections before making your selection.

☒ Life sciences ☐ Behavioural & social sciences ☐ Ecological, evolutionary & environmental sciences

For a reference copy of the document with all sections, see nature.com/documents/nr-reporting-summary-flat.pdf

Life sciences study design

All studies must disclose on these points even when the disclosure is negative.

Sample size	For the sequencing experiments, n = 4 per condition was chosen to maximize the data while minimizing animal and monetary cost. No statistical tests were utilized for determining sample size. Sample sizes were estimated based on preliminary experiments. All sample sizes are provided in the figure legends.
Data exclusions	No data exclusion was performed
Replication	All the experiments were replicated. The number of replicates is stated in figure legends.
Randomization	Animals of the indicated ages were randomized in the described groups in each analysis
Blinding	Histology quantification was evaluated by pathologists blinded to the experimental conditions. No blinding was applied for other experiments.

Reporting for specific materials, systems and methods

We require information from authors about some types of materials, experimental systems and methods used in many studies. Here, indicate whether each material, system or method listed is relevant to your study. If you are not sure if a list item applies to your research, read the appropriate section before selecting a response.

Materials & experimental systems

n/a	Involved in the study	
☐	☒	Antibodies
☒	☐	Eukaryotic cell lines
☒	☐	Palaeontology and archaeology
☐	☒	Animals and other organisms
☒	☐	Clinical data
☒	☐	Dual use research of concern
☒	☐	Plants

Methods

n/a	Involved in the study	
☒	☐	ChIP-seq
☐	☒	Flow cytometry
☒	☐	MRI-based neuroimaging

Antibodies

Antibodies used

Antibodies used in flow cytometry:
CD16/32 (clone 2.4G2, Biolegend), CD11b (clone M1/70, Thermo Fisher Scientific), F4/80 (clone BM8, Biolegend), Flag-tag (clone L5, Biolegend), CD90.1 (clone HIS51, Thermo Fisher Scientific). pSTAT6 (clone CHI2S4N, Thermo Fisher Scientific), pAKT (J1-223.371, BD Biosciences), pJAK3 (JAK3Y980981-E10, Thermo Fisher Scientific), pTYK2 (Thermo Fisher Scientific), CD206 (clone C068C2, Biolegend), EGR2 (clone Erongr2, Thermo Fisher Scientific), PD-L2 (clone TY25, Thermo Fisher Scientific), CD86 (clone GL1, Biolegend) and CD38 (clone 90, Thermo Fisher Scientific), anti-CD45 antibody (clone 30-F11, Biolegend), CD3 (clone 145-2C11, Biolegend), CD4 (clone GK1.5, Biolegend), Foxp3 (FJK-16s, Thermo Fisher Scientific), CD8a (clone 53-6.7, Biolegend), Granzyme B (clone NGZB, Thermo Fisher Scientific), Perforin (clone eBioOMAK-D, Thermo Fisher Scientific), CD14 (M5E2, Biolegend), CD80 (2D10.4, Biolegend), CD86 (IT2, Thermo Fisher Scientific), CD206 (19.2, Thermo Fisher Scientific), CD200R (OX108, Thermo Fisher Scientific)
Antibodies used in immunofluorescence experiments:
CD45.1 (clone A20, Biolegend), CD90.1 (clone HIS51, Thermo Fisher Scientific), CD206 (clone C068C2, Biolegend), Gr-1 (clone Ly-6G, Biolegend) and Ki67 (clone 11F6, Biolegend).

Validation

All antibodies were well-recognized clones in the field and validated by the manufacturers.

Animals and other research organisms

Policy information about studies involving animals; ARRIVE guidelines recommended for reporting animal research, and Sex and Gender in Research

Laboratory animals

Eight- to twelve-week-old male BALB/c, C57BL/6 (CD45.2+), congenic C57BL/6 (CD45.1+) and TNFα-/- (B6 · 129S-Tnftm1Gkl/J) mice were purchased from the Australian BioResources (Sydney, Australia). All mice were maintained under SPF housing with a maximum of four mice per cage.

Wild animals

The study did not involve wild animals.

Reporting on sex

Male C57BL/6 mice were exclusively employed to induce kidney and liver ischemia-reperfusion injury (IRI) due to the observation that female C57BL/6 mice typically exhibit only mild tissue injury in the kidney or liver following IRI.
The strain of inbred animal is important for the induction of Adriamycin nephrosis (AN). AN occurs only in certain strains such as male BALB/c mice, male Wistar rats, whilst other strains (such as C57BL/6) are relatively resistant.

Field-collected samples

No field-collected samples were used.

Ethics oversight

All experiments with mice were performed in accordance with protocols approved by Animal Ethics Committee of Western Sydney Local Health District (WSLHD).

Note that full information on the approval of the study protocol must also be provided in the manuscript.

Plants

Seed stocks

Report on the source of all seed stocks or other plant material used. If applicable, state the seed stock centre and catalogue number. If plant specimens were collected from the field, describe the collection location, date and sampling procedures.

Novel plant genotypes

Describe the methods by which all novel plant genotypes were produced. This includes those generated by transgenic approaches, gene editing, chemical/radiation-based mutagenesis and hybridization. For transgenic lines, describe the transformation method, the number of independent lines analyzed and the generation upon which experiments were performed. For gene-edited lines, describe the editor used, the endogenous sequence targeted for editing, the targeting guide RNA sequence (if applicable) and how the editor was applied.

Authentication

Describe any authentication procedures for each seed stock used or novel genotype generated. Describe any experiments used to assess the effect of a mutation and, where applicable, how potential secondary effects (e.g. second site T-DNA insertions, mosiacism, off-target gene editing) were examined.

Flow Cytometry

Plots

Confirm that:

☒ The axis labels state the marker and fluorochrome used (e.g. CD4-FITC).

☒ The axis scales are clearly visible. Include numbers along axes only for bottom left plot of group (a 'group' is an analysis of identical markers).

☒ All plots are contour plots with outliers or pseudocolor plots.

☒ A numerical value for number of cells or percentage (with statistics) is provided.

Methodology

Sample preparation	Primary mouse and human macrophages differentiated in culture were detached from flasks or dishes and counted prior to staining. At the time of sacrifice, animals were transcardially perfused with 1xPBS until the kidney, liver and lung demonstrated clearing. Spleen was isolated, minced, and digested for 30 min at 37? in RPMI 1640 containing 1 mg/ml collagenase D (Roche) and 100 µg/ml DNase I (Roche). The digested cell suspension was then passed through a 40-µm cell strainer. Kidney, liver and lung were perfused with saline before removal and digested with collagenase and DNase as previously described. Kidney, liver and lung were cut into 1 to 2 mm3 pieces, placed in DMEM containing 1 mg/ml collagenase IV (Sigma Aldrich), and 100 µg/ml DNase I (Roche) for 40 minutes at 37? with intermittent agitation. The digested cell suspension was then passed through a 40-µm cell strainer. The transfused CAR-Ms or UTD-Ms were sorted from various organs by FACS for further analysis.
Instrument	BD LSRFortessa and FACSAria II
Software	FACS DIVA software v8. For data analysis, FlowJo TM V10
Cell population abundance	When cells were sorted or enriched, the purity was confirmed by flow cytometry and in each case the purity was above 90%.
Gating strategy	The cells were gated on FSC-A/SSC-A basis on the location known to contain lymphocytes and myeloid cells. Doublets were excluded based on FSC-A/FSC-H gating. non-viable cells were excluded by staining with DAPI or 7-AAD. CD45+ cells were gated for analysis of either T cells or myeloid cells. Transfused UTD-Ms and CAR-Ms were gated on CD45.1+F4/80+ cells or CD90.1+F4/80+ cells. Endogenous Treg cells were gated on CD45.2+CD3+CD4+Foxp3+ cells. Endogenous CD8+ T cells and macrophages were gated on CD45.2+CD3+CD8+ and CD45.2+CD11b+F4/80+ analyzed for phenotype.

☒ Tick this box to confirm that a figure exemplifying the gating strategy is provided in the Supplementary Information.
